# ISSN exercise & sport nutrition review: research & recommendations

**DOI:** 10.1186/1550-2783-7-7

**Published:** 2010-02-02

**Authors:** Richard B Kreider, Colin D Wilborn, Lem Taylor, Bill Campbell, Anthony L Almada, Rick Collins, Mathew Cooke, Conrad P Earnest, Mike Greenwood, Douglas S Kalman, Chad M Kerksick, Susan M Kleiner, Brian Leutholtz, Hector Lopez, Lonnie M Lowery, Ron Mendel, Abbie Smith, Marie Spano, Robert Wildman, Darryn S Willoughby, Tim N Ziegenfuss, Jose Antonio

**Affiliations:** 1 Exercise & Sports Nutrition Lab, Texas A&M University, College Station, TX, USA; 2 Exercise & Sport Science Department, University of Mary-Hardin Baylor, Belton, TX, USA; 3 School of Physical Education & Exercise Science, University of South Florida, Tampa, FL, USA; 4 GENr8, Inc, Dana Point, CA, USA; 5 Collins, McDonald & Gann, PC, Mineola, NY, USA; 6 Schools of Medicine & Health Movement Studies, The University of Queensland, Herston, Queensland, AU; 7 Pennington Biomedical Reseach Center, Baton Rouge, LA, USA; 8 Department of Health, Human Performance, and Recreation, Baylor University, Box 97313, Waco, TX, USA; 9 Miami Research Associates, Miami, FL, USA; 10 Department of Health and Exercise Science, University of Oklahoma, Norman, OK, USA; 11 High Performance Nutrition LLC, Mercer Island, WA, USA; 12 Northwestern University Feinberg School of Medicine, Department of Physical Medicine and Rehabilitation, Rehabilitation Institute of Chicago, Chicago, IL, USA; 13 Nutrition Assessment Laboratory, Nutrition Center, University of Akron, Akron, Ohio, USA; 14 Department of Human Performance & Sport Management, Mount Union College, Alliance, Ohio, USA; 15 Marie Spano Nutrition Consulting, Atlanta, GA, USA; 16 Department of Human Nutrition, Kansas State University, Manhattan KS, USA; 17 Division of Sports Nutrition and Exercise Science, the Center for Applied Health Sciences, Fairlawn, OH, USA; 18 Farquhar College of Arts and Sciences, Nova Southeastern University, Fort Lauderdale, FL, USA

## Abstract

Sports nutrition is a constantly evolving field with hundreds of research papers published annually. For this reason, keeping up to date with the literature is often difficult. This paper is a five year update of the sports nutrition review article published as the lead paper to launch the JISSN in 2004 and presents a well-referenced overview of the current state of the science related to how to optimize training and athletic performance through nutrition. More specifically, this paper provides an overview of: 1.) The definitional category of ergogenic aids and dietary supplements; 2.) How dietary supplements are legally regulated; 3.) How to evaluate the scientific merit of nutritional supplements; 4.) General nutritional strategies to optimize performance and enhance recovery; and, 5.) An overview of our current understanding of the ergogenic value of nutrition and dietary supplementation in regards to weight gain, weight loss, and performance enhancement. Our hope is that ISSN members and individuals interested in sports nutrition find this review useful in their daily practice and consultation with their clients.

## Introduction

Sports nutrition professionals need to know how to evaluate the scientific merit of articles and advertisements about exercise and nutrition products so they can separate marketing hype from scientifically-based training and nutritional practices. In order to help ISSN members keep informed about the latest in sports nutrition, we have updated the ISSN Exercise & Sports Nutrition Review that was used to help launch the JISSN (originally called the Sports Nutrition Review Journal). This paper provides an overview of: 1.) The definitional category of ergogenic aids and dietary supplements; 2.) How dietary supplements are legally regulated; 3.) How to evaluate the scientific merit of nutritional supplements; 4.) General nutritional strategies to optimize performance and enhance recovery; and, 5.) An overview of our current understanding of the ergogenic value in regards to weight gain, weight loss, and performance enhancement supplements. We have also categorized nutritional supplements into 'apparently effective', 'possibly effective', 'too early to tell', and 'apparently ineffective' as well a description of our general approach into educating athletes about sports nutrition. Over the last five years there have been many changes to our original categorization of supplements. In addition, a number of new supplements have been introduced to the market are reviewed in this article. While some may not agree with all of our interpretations of the literature and/or categorization of a particular supplement, and some classifications may change over time as more research is forthcoming, these interpretations are based on current available scientific evidence and have been well received within the broader scientific community. Our hope is that ISSN members find this information useful in their daily practice and consultation with their clients.

## Ergogenic Aid

An ergogenic aid is any training technique, mechanical device, nutritional practice, pharmacological method, or psychological technique that can improve exercise performance capacity and/or enhance training adaptations [1-3]. This includes aids that may help prepare an individual to exercise, improve the efficiency of exercise, and/or enhance recovery from exercise. Ergogenic aids may also allow an individual to tolerate heavy training to a greater degree by helping them recover faster or help them stay injury-free and/or healthy during intense training. Although this definition seems rather straightforward, there is considerable debate regarding the ergogenic value of various nutritional supplements. Some sports nutrition specialists only consider a supplement ergogenic if studies show that the supplement significantly enhances exercise performance (e.g., helps you run faster, lift more weight, and/or perform more work during a given exercise task). On the other hand, some feel that if a supplement helps prepare an athlete to perform or enhances recovery from exercise, it has the potential to improve training adaptations and therefore should be considered ergogenic. In the view of the ISSN, one should take a broader view about the ergogenic value of supplements. While we are interested in determining the performance enhancement effects of a supplement on a single bout of exercise, we also realize that one of the goals of training is to help people tolerate a greater degree of training. Individuals who better adapt to high levels of training usually experience greater gains from training over time which can lead to improved performance. Consequently, employing nutritional practices that help prepare individuals to perform and/or enhance recovery from exercise should also be viewed as ergogenic.

## Definition and Regulation of Dietary Supplements

As described in Exercise and Sports Nutrition: Principles, Promises, Science & Recommendations [3]; according to the Food and Drug Administration (FDA), dietary supplements were regulated in the same manner as food prior to 1994 [4]. Consequently, the FDA monitored the manufacturing processes, quality, and labeling of dietary supplements. However, many people felt that the FDA was too restrictive in regulating dietary supplements. As a result, Congress passed the Dietary Supplement Health and Education Act (DSHEA) in 1994 which placed dietary supplements in a special category of "foods". In October 1994, President Clinton signed DSHEA into law. The law defined a "dietary supplement" as a product taken by mouth that contains a "dietary ingredient" intended to supplement the diet. "Dietary ingredients" may include vitamins, minerals, herbs or other botanicals, amino acids, and substances (e.g., enzymes, organ tissues, glandular, and metabolites). Dietary supplements may also be extracts or concentrates from plants or foods. Dietary supplements are typically sold in the form of tablets, capsules, soft gels, liquids, powders, and bars. Products sold as dietary supplements must be clearly labeled as a dietary supplement.

According to DSHEA, dietary supplements are not drugs. Dietary supplement ingredients that were lawfully sold prior to 1994, have been "grandfathered" into the Act, meaning that a manufacturer is not required to submit to FDA the evidence it relies upon to substantiate safety or effectiveness before or after it markets these ingredients. The rationale for this exclusion is based on a long history of safe use; hence there is no need to require additional safety data. However, DSHEA grants FDA greater control over supplements containing new dietary ingredients. A new dietary ingredient is deemed adulterated and subject to FDA enforcement sanctions unless it meets one of two exemption criteria: either 1.) the supplement in question contains "only dietary ingredients which have been present in the food supply as an article used for food in a form in which the food has not been chemically altered"; or 2.) there is a "history of use or other evidence of safety" provided by the manufacturer or distributor to FDA at least 75 days before introducing the product into interstate commerce. The second criterion, applicable only to new dietary ingredients that have not been present in the food supply, requires manufacturers and distributors of a new dietary ingredient or a product containing a new dietary ingredient to submit pre-market notification to the FDA. This notification, which must be submitted at least 75 days before the product is introduced into interstate commerce, must contain information that provides a history of use or other evidence of safety establishing that the dietary ingredient, when used under the conditions recommended or suggested in the labeling of the dietary supplement will "reasonably be expected to be safe." This may include conducting in vitro toxicology testing, long-term toxicity studies using varying doses in animals to see if there are any toxic effects, providing manufacturing and quality assurance data showing purity, and provision of clinical studies conducted in humans showing safety. The FTC also requires that any representations or claims made about the supplement be substantiated by adequate evidence to show that they are not false or misleading, a policy which is also shared by the FDA. This involves, for example, providing at least two clinical trials showing efficacy of the actual product, within a population of subjects relevant to the target market, supporting the structure/function claims that are made. Structure/function claims may include several categories. They may describe the role of a nutrient or dietary ingredient intended to affect normal structure or function in humans, they may characterize the means by which a nutrient or dietary ingredient acts to maintain such structure or function, they may describe general well-being from consumption of a nutrient or dietary ingredient or they may describe a benefit related to a nutrient deficiency disease, as long as the statement also tells how widespread such a disease is in the United States. Manufacturers of dietary supplements that make structure/function claims on labels or in labeling must submit a notification to FDA no later than 30 days after marketing the dietary supplement that includes the text of the structure/function claim. DSHEA also requires supplement manufacturers to include on any label displaying structure/function claims the disclaimer *"This statement has not been evaluated by the FDA. This product is not intended to diagnose, treat, cure, or prevent any disease"*. Opponents of dietary supplements often cite this statement as evidence that the FDA did not review or approve the dietary supplement when in fact most dietary ingredients have been grandfathered in due to a long history of safe sale; whereas those products containing a new dietary ingredient which is not present in the food supply as an article used for food in a form in which the food has not been chemically altered are subject to pre-market notification to FDA regarding history of use or other evidence of safety. Unfortunately, a large number of new dietary ingredients requiring pre-market notification have been introduced into dietary supplements since October 1994 without the requisite notification.

According to the 1994 Nutrition Labeling and Education Act (NLEA), the FDA has the ability to review and approve health claims for dietary ingredients and foods. However, since the law was passed it has only approved a few claims. The delay in reviewing health claims of dietary supplements resulted in a lawsuit filed by **Pearson & Shaw et al v. Shalala **et al in 1993. After years of litigation, the U.S. Court of Appeals for the District of Columbia Circuit ruled in 1999 that qualified health claims may now be made about dietary supplements with approval by FDA as long as the statements are truthful and based on science. Supplement or food companies wishing to make health claims about supplements can submit research evidence to the FDA for approval of a health claim. Additionally, companies must also submit an Investigational New Drug (IND) application to FDA if a research study on a nutrient or multiple dietary ingredient composition is designed to treat an illness and/or medical affliction and/or the company hopes to one day obtain approval for making a qualified health claim as a prescription or orphan drug if the outcome of the study supports the claim. Studies investigating structure/function claims, however, do not need to be submitted to the FDA as an IND. The 1997 Food and Drug Administration Modernization Act (FDAMA) provided for health claims based on an authoritative statement of a scientific body of the U.S. Government or the National Academy of Sciences; such claims may be used after submission of a health claim notification to FDA; and the 2003 FDA *Consumer Health Information for Better Nutrition Initiative *provided for qualified health claims where the quality and strength of the scientific evidence falls below that required for FDA to issue an authorizing regulation. Such health claims must be qualified to assure accuracy and non-misleading presentation to consumers. More recently, the U.S. Senate passed legislation (Senate Bill 1082) that established the Reagan-Udall Foundation for the FDA. The purpose of this non-profit foundation is to lead collaborations among the FDA, academic research institutions, and industry to enhance research in evaluating the safety and efficacy of dietary supplements as well as to improve the quality and management of these products.

For many years, manufacturers and distributors of dietary supplements were not required to record, investigate or forward to FDA any reports they receive on injuries or illnesses that may be related to the use of their products. However, companies are now required by the Dietary and Supplement and Nonprescription Drug Consumer Act (Public Law 109-462 109th Congress Dec. 22, 2006) to record all adverse event complaints about their products and make them available to the FDA pursuant to an inspection. Reports of "serious" adverse events (i.e., adverse events which results in death, a life-threatening experience, inpatient hospitalization, a persistent or significant disability or incapacity, or a congenital anomaly or birth defect; or requires, based on a reasonable medical judgment, a medical or surgical intervention to prevent an outcome described above) must be reported to FDA within 15 business days. While these reports are unsubstantiated; can be influenced by media attention to a particular supplement; and do not necessarily show a cause and effect: they can be used by the company and FDA to monitor trends and "signals" that may suggest a problem. Once a dietary supplement product is marketed, the FDA has the responsibility for showing that the dietary supplement is unsafe before it can take action to restrict the product's use or removal from the marketplace. The FTC maintains responsibility to make sure manufacturers are truthful and not misleading regarding claims they make about dietary supplements. The FDA has the power to remove supplements from the market if it has sufficient scientific evidence to show the supplement is unsafe. Once they do, they must have sufficient evidence to meet review by the Office of General Accounting (OGA) and/or legal challenges. In the past, the FDA has acted to remove dietary supplements from the market only to be concluded by the OGA and/or federal courts to have overstepped their authority. Additionally, the FTC has the power to act against companies who make false and/or misleading marketing claims about a specific product. This includes acting against companies if the ingredients found in the supplement do not match label claims or in the event undeclared, drug ingredients are present (e.g., analogs of weight loss drugs, diuretic drugs). While this does not ensure the safety of dietary supplements, it does provide a means for governmental oversight of the dietary supplement industry if adequate resources are provided to enforce DSHEA. Since the inception of DSHEA, the FDA has required a number of supplement companies to submit evidence showing safety of their products and acted to remove a number of products sold as dietary supplements from sale in the United States due to safety concerns. Additionally, the FTC has acted against a number of supplement companies for misleading advertisements and/or structure and function claims.

As demonstrated, while some argue that the dietary supplement industry is "unregulated" and/or may have suggestions for additional regulation, manufacturers of dietary supplements must adhere to a number of federal regulations before a product can go to market. Further, they must have evidence that the ingredients sold in their supplements are generally safe if requested to do so by the FDA. For this reason, over the last 20 years, a number of quality supplement companies have employed research and development directors who help educate the public about nutrition and exercise, provide input on product development, conduct preliminary research on products, and/or assist in coordinating research trials conducted by independent research teams (e.g., university based researchers or clinical research sites). They also consult with marketing and legal teams with the responsibility to ensure structure and function claims do not misrepresent results of research findings. This has increased job opportunities for sports nutrition specialists as well as enhanced external funding opportunities for research groups interested in exercise and nutrition research.

While it is true that a number of companies falsely attribute research on different dietary ingredients or dietary supplements to their own, suppress negative findings, and/or exaggerate results from research studies; the trend in the nutrition industry has been to develop scientifically sound supplements. This trend toward greater research support is the result of: 1.) Attempts to honestly and accurately inform the public about results; 2.) Efforts to have data to support safety and efficacy on products for FDA and the FTC; and/or, 3.) To provide scientific evidence to support advertising claims and increase sales. This trend is due in part to greater scrutiny from the FDA and FTC, but also in response to an increasingly competitive marketplace where established safety and efficacy attracts more consumer loyalty and helps ensure a longer lifespan for the product in commerce. In our experience, companies who adhere to these ethical standards prosper while those who do not struggle to comply with FDA and FTC guidelines and rapidly lose consumer confidence, signaling an early demise for the product.

## Product Development and Quality Assurance

One of the most common questions raised by athletes, parents, and professionals regarding dietary supplements relates to how they are manufactured and consumer awareness of supplement quality. In a number of cases, reputable companies who develop dietary supplements have research teams who scour the medical and scientific literature looking for potentially effective nutrients. These research teams often attend scientific meetings and review the latest patents, research abstracts presented at scientific meetings, and research publications. They may also consult with leading researchers to discuss ideas about dietary supplements that can be commercialized. Leading companies invest in basic research on nutrients before developing their supplement formulations. Others wait until research has been presented in patents, research abstracts, or publications before developing nutritional formulations featuring the nutrient. Once a new nutrient or formulation has been identified, the next step is to contact raw ingredient suppliers to see if the nutrient can be obtained in a highly pure source and/or if it's affordable. Sometimes, companies develop and patent new processing and purification processes because the nutrient has not yet been extracted in a pure form or is not available in large quantities. Reputable raw material manufacturers conduct extensive tests to examine purity of their raw ingredients. If the company is working on a new ingredient, they often conduct toxicity studies on the new nutrient once a purified source has been identified. They would then compile a safety dossier and communicate it to the FDA as a New Dietary Ingredient submission, with the hopes of it being allowed for lawful sale.

When a powdered formulation is designed, the list of ingredients and raw materials are typically sent to a flavoring house and packaging company to identify the best way to flavor and package the supplement. In the nutrition industry, there are several main flavoring houses and packaging companies who make a large number of dietary supplements for dietary supplement companies. Most reputable dietary supplement manufacturers submit their production facilities to inspection from the FDA and adhere to good manufacturing practices (GMP's), which represent industry standards for good manufacturing of dietary supplements. Some companies also submit their products for independent testing by third-party companies to certify that their products meet label claims. For example, NSF's certification service includes product testing, GMP inspections, ongoing monitoring and use of the NSF Mark indicating products comply with inspection standards, and screening for contaminants. More recently, companies have subjected their products for testing by third party companies to inspect for banned or unwanted substances. These types of tests help ensure that each batch of the dietary supplement does not contained substances banned by the International Olympic Committee or other athletic governing bodies (e.g., NFL). While third-party testing does not guarantee that a supplement is void of banned substances, the likelihood is much less (e.g., Banned Substances Control Group, Informed Choice, etc). Moreover, consumers can request copies of results of these tests. In our experience, companies who are not willing to provide copies of test results are not worth purchasing.

## Evaluation of Nutritional Ergogenic Aids

The ISSN recommends going through a process of evaluating the validity and scientific merit of claims made when assessing the ergogenic value of a dietary supplement/technique [3]. This can be accomplished by examining the theoretical rationale behind the supplement/technique and determining whether there is any well-controlled data showing the supplement/technique works. Supplements based on sound scientific rationale with direct, supportive research showing effectiveness may be worth trying and/or recommending. However, those based on unsound scientific results and/or little to no data supporting the ergogenic value of the actual supplement/technique may not be worthwhile. The sports nutrition specialist should be a resource to help their clients interpret the scientific and medical research that may impact their welfare and/or help them train more wisely and effectively. The following are recommended questions to ask when evaluating the potential ergogenic value of a supplement.

### Does The Theory Make Sense?

Most supplements that have been marketed to improve health and/or exercise performance are based on theoretical applications derived from basic and/or clinical research studies. Based on these preliminary studies, a training device or supplement is often marketed to people proclaiming the benefits observed in these basic research studies. Although the theory may appear relevant, critical analysis of this process often reveals flaws in scientific logic and/or that the claims made don't quite match up with the literature cited. By evaluating the literature on your own you can discern whether a supplement has been based on sound scientific evidence or not. To do so, it is suggested you read reviews about the training method, nutrient, and/or supplement from researchers who have been intimately involved in this line of research and/or consult reliable references about nutritional and herbal supplements, such as the JISSN [3,5]. We also suggest doing a search on the nutrient/supplement on the National Library of Medicine's Pub Med Online http://www.ncbi.nlm.nih.gov. A quick look at these references will often help determine if the theory is plausible or not. In our experience, proponents of ergogenic aids often overstate claims made about training devices and/or dietary supplements while opponents of dietary supplements and ergogenic aids are either unaware and/or ignorant of research supporting their use. The sports nutrition specialist has the responsibility to know the literature and/or search available databases to evaluate whether there is merit or not to a proposed ergogenic aid.

### Is There Any Scientific Evidence Supporting The Ergogenic Value?

The next question to ask is whether there is any well-controlled data showing effectiveness of the proposed ergogenic aid works as claimed in athletes or people involved in training. The first place to look is the list of references cited in marketing material supporting their claims. We look to see if the abstracts or articles cited are general references or specific studies that have evaluated the efficacy of the nutrient/supplement. We then critically evaluate the abstracts and articles by asking a series of questions.

• Are the studies basic research done in animals/clinical populations or have the studies been conducted on athletes/trained subjects? Studies reporting improved performance in rats or persons with type 2 diabetes may be insightful but research conducted on non-diabetic athletes is much more practical and relevant.

• Were the studies well controlled? For ergogenic aid research, the study should be a placebo controlled, double-blind, and randomized clinical trial if possible. This means that neither the researcher's nor the subject's were aware which group received the supplement or the placebo during the study and that the subjects were randomly assigned into the placebo or supplement group. An additional element of rigor is called a cross-over design, where each subject, at different times (separated by an interval known as a "washout period"), is exposed to each of the treatments. While utilization of a cross-over design is not always feasible, it removes the element of variability between subjects and increases the strength of the findings. At times, supplement claims have been based on poorly designed studies (i.e., small groups of subjects, no control group, use of unreliable tests, etc) and/or testimonials which make interpretation much more difficult. Well-controlled clinical trials provide stronger evidence as to the potential ergogenic value.

• Do the studies report statistically significant results or are claims being made on non-significant means or trends reported? Appropriate statistical analysis of research results allows for an unbiased interpretation of data. Although studies reporting statistical trends may be of interest and lead researchers to conduct additional research, studies reporting statistically significant results are obviously more convincing. With this said, a sports nutrition specialist must be careful not to commit type II statistical errors (i.e., indicating that no differences were observed when a true effect was seen but not detected statistically). Since many studies on ergogenic aids (particularly in high level athletes) evaluate small numbers of subjects, results may not reach statistical significance even though large mean changes were observed. In these cases, additional research is warranted to further examine the potential ergogenic aid before conclusions can be made.

• Do the results of the studies cited match the claims made about the supplement? It is not unusual for marketing claims to greatly exaggerate the results found in the actual studies. Additionally, it is not uncommon for ostensibly compelling results, that may indeed by statistically significant, to be amplified while other relevant findings of significant consumer interest are obscured or omitted (e.g. a dietary supplement showing statistically significant increases in circulating testosterone yet changes in body composition or muscular performance were not superior to a placebo). The only way to determine this is to read the entire article, and not just the abstract or even the article citation, and compare results observed in the studies to marketing claims. Reputable companies accurately and completely report results of studies so that consumers can make informed decisions about whether to try a product or not.

• Were results of the study presented at a reputable scientific meeting and/or published in a peer-reviewed scientific journal? At times, claims are based on research that has either never been published or only published in an obscure journal. The best research is typically presented at respected scientific meetings and/or published in reputable peer-reviewed journals. Two ways to determine a journal's reputation is either identifying the publisher or the "impact factor" of the journal. A number of "peer-reviewed" journals are published by companies with ties to, or are actually owned by, nutritional products companies (even though they may be available on PubMed). Therefore, we recommend looking up the publisher's website and see how many other journals they publish. If you see only a few other journals this is a suggestion that the journal is not a reputable journal. Alternatively, inquire about the impact factor, a qualitative ranking determined by the number of times a journal's articles are cited. Impact factors are determined and published by Thomson Reuters under Journal Citation Reports^® ^(a subscription service available at most university libraries). Most journals list their impact factor on the journal home page. The most significant and erudite scientific articles are typically the most read and the most cited.

• Have the research findings been replicated at several different labs? The best way to know an ergogenic aid works is to see that results have been replicated in several studies preferably by a number of separate, distinct research groups. The most reliable ergogenic aids are those in which a number of studies, conducted at different labs, have reported similar results of safety and efficacy. Additionally, replication of results by different, unaffiliated labs with completely different authors also removes or reduces the potentially confounding element of publication bias (publication of studies showing only positive results) and conflicts of interest. A notable number of studies on ergogenic aids are conducted in collaboration with one or more research scientists or co-investigators that have a real or perceived economic interest in the outcome of the study. This could range from being a co-inventor on a patent application that is the subject of the ergogenic aid, being paid or receiving royalties from the creation of a dietary supplement formulation, or having stock options or shares in a company that owns or markets the ergogenic aid described in the study. An increasing number of journals require disclosures by all authors of scientific articles, and including such disclosures in published articles. This is driven by the aim of providing greater transparency and research integrity. Disclosure of a conflict of interest does not alone discredit or dilute the merits of a research study. The primary thrust behind public disclosures of potential conflicts of interest is the prevention of a later revelation of an interest that has the potential of discrediting the study in question, the authors, and even the research center or institution where the study was conducted.

### Is The Supplement Legal And Safe?

The final question that should be asked is whether the supplement is legal and/or safe. Some athletic associations have banned the use of various nutritional supplements (e.g., prohormones, Ephedra that contains ephedrine, "muscle building" supplements, etc). Obviously, if the supplement is banned, the sports nutrition specialist should discourage its use. In addition, many supplements have not been studied for long-term safety. People who consider taking nutritional supplements should be well aware of the potential side effects so that they can make an informed decision regarding whether to use a supplement or not. Additionally, they should consult with a knowledgeable physician to see if there are any underlying medical problems that may contraindicate use. When evaluating the safety of a supplement, we suggest looking to see if any side effects have been reported in the scientific or medical literature. In particular, we suggest determining how long a particular supplement has been studied, the dosages evaluated, and whether any side effects were observed. We also recommend consulting the Physician's Desk Reference (PDR) for nutritional supplements and herbal supplements to see if any side effects have been reported and/or if there are any known drug interactions. If no side effects have been reported in the scientific/medical literature, we generally will view the supplement as safe for the length of time and dosages evaluated.

## Classifying and Categorizing Supplements

Dietary supplements may contain carbohydrate, protein, fat, minerals, vitamins, herbs, enzymes, metabolic intermediates (like amino acids), and/or various plant/food extracts. Supplements can generally be classified as convenience supplements (e.g., energy bars, meal replacement powders, ready to drink supplements) designed to provide a convenient means of meeting caloric needs and/or managing caloric intake, weight gain, weight loss, and/or performance enhancement. Based on the above criteria, we generally categorize nutritional supplements into the following categories:

I. **Apparently Effective**. Supplements that help people meet general caloric needs and/or the majority of research studies in relevant populations show is effective and safe.

II. **Possibly Effective**. Supplements with initial studies supporting the theoretical rationale but requiring more research to determine how the supplement may affect training and/or performance.

III. **Too Early To Tell**. Supplements with sensible theory but lacking sufficient research to support its current use.

IV. **Apparently Ineffective**. Supplements that lack a sound scientific rationale and/or research has clearly shown to be ineffective.

When a sports nutrition specialist counsels people who train, they should first evaluate their diet and training program. They should make sure that the athlete is eating an energy balanced, nutrient dense diet and that they are training intelligently. This is the foundation to build a good program. Following this, we suggest that they generally only recommend supplements in category I (i.e., 'Apparently Effective). If someone is interested in trying supplements in category II (i.e., 'Possibly Effective'), they should make sure that they understand that these supplements are more experimental and that they may or may not see the type of results claimed. We recommend discouraging people from trying supplements in category III (i.e., 'Too Early to Tell') because there isn't enough data available on their ergogenic value. However, if someone wants to try one of these supplements, they should understand that although there is some theoretical rationale, there is little evidence to support use at this time. Obviously, we do not support athletes taking supplements in categories IV (i.e., 'Apparently Ineffective'). We believe that this approach is a more scientifically supportable and balanced view than simply dismissing the use of all dietary supplements out of hand.

## General Dietary Guidelines for Active Individuals

A well-designed diet that meets energy intake needs and incorporates proper timing of nutrients is the foundation upon which a good training program can be developed. Research has clearly shown that not ingesting a sufficient amount of calories and/or enough of the right type of macronutrients may impede an athlete's training adaptations while athletes who consume a balanced diet that meets energy needs can augment physiological training adaptations. Moreover, maintaining an energy deficient diet during training may lead to loss of muscle mass and strength, increased susceptibility to illness, and increased prevalence of overreaching and/or overtraining. Incorporating good dietary practices as part of a training program is one way to help optimize training adaptations and prevent overtraining. The following overviews energy intake and major nutrient needs of active individuals.

### Energy Intake

The first component to optimize training and performance through nutrition is to ensure the athlete is consuming enough calories to offset energy expenditure [1,6-8]. People who participate in a general fitness program (e.g., exercising 30 - 40 minutes per day, 3 times per week) can typically meet nutritional needs following a normal diet (e.g., 1,800 - 2,400 kcals/day or about 25 - 35 kcals/kg/day for a 50 - 80 kg individual) because their caloric demands from exercise are not too great (e.g., 200 - 400 kcals/session) [1]. However, athletes involved in moderate levels of intense training (e.g., 2-3 hours per day of intense exercise performed 5-6 times per week) or high volume intense training (e.g., 3-6 hours per day of intense training in 1-2 workouts for 5-6 days per week) may expend 600 - 1,200 kcals or more per hour during exercise [1,9]. For this reason, their caloric needs may approach 50 - 80 kcals/kg/day (2,500 - 8,000 kcals/day for a 50 - 100 kg athlete). For elite athletes, energy expenditure during heavy training or competition may be enormous. For example, energy expenditure for cyclists to compete in the Tour de France has been estimated as high as 12,000 kcals/day (150 - 200 kcals/kg/d for a 60 - 80 kg athlete) [9-11]. Additionally, caloric needs for large athletes (i.e., 100 - 150 kg) may range between 6,000 - 12,000 kcals/day depending on the volume and intensity of different training phases [9].

Although some argue that athletes can meet caloric needs simply by consuming a well-balanced diet, it is often very difficult for larger athletes and/or athletes engaged in high volume/intense training to be able to eat enough food in order to meet caloric needs [1,7,9,10,12]. Maintaining an energy deficient diet during training often leads to significant weight loss (including muscle mass), illness, onset of physical and psychological symptoms of overtraining, and reductions in performance [8]. Nutritional analyses of athletes' diets have revealed that many are susceptible to maintaining negative energy intakes during training. Susceptible populations include runners, cyclists, swimmers, triathletes, gymnasts, skaters, dancers, wrestlers, boxers, and athletes attempting to lose weight too quickly [7]. Additionally, female athletes have been reported to have a high incidence of eating disorders [7]. Consequently, it is important for the sports nutrition specialist working with athletes to ensure that athletes are well-fed and consume enough calories to offset the increased energy demands of training, and maintain body weight. Although this sounds relatively simple, intense training often suppresses appetite and/or alters hunger patterns so that many athletes do not feel like eating [7]. Some athletes do not like to exercise within several hours after eating because of sensations of fullness and/or a predisposition to cause gastrointestinal distress. Further, travel and training schedules may limit food availability and/or the types of food athletes are accustomed to eating. This means that care should be taken to plan meal times in concert with training, as well as to make sure athletes have sufficient availability of nutrient dense foods throughout the day for snacking between meals (e.g., drinks, fruit, carbohydrate/protein bars, etc) [1,6,7]. For this reason, sports nutritionists' often recommend that athletes consume 4-6 meals per day and snacks in between meals in order to meet energy needs. Use of nutrient dense energy bars and high calorie carbohydrate/protein supplements provides a convenient way for athletes to supplement their diet in order to maintain energy intake during training.

### Carbohydrate

The second component to optimizing training and performance through nutrition is to ensure that athletes consume the proper amounts of carbohydrate (CHO), protein (PRO) and fat in their diet. Individuals engaged in a general fitness program can typically meet macronutrient needs by consuming a normal diet (i.e., 45-55% CHO [3-5 grams/kg/day], 10-15% PRO [0.8 - 1.0 gram/kg/day], and 25-35% fat [0.5 - 1.5 grams/kg/day]). However, athletes involved in moderate and high volume training need greater amounts of carbohydrate and protein in their diet to meet macronutrient needs. For example, in terms of carbohydrate needs, athletes involved in moderate amounts of intense training (e.g., 2-3 hours per day of intense exercise performed 5-6 times per week) typically need to consume a diet consisting of 55-65% carbohydrate (i.e., 5-8 grams/kg/day or 250 - 1,200 grams/day for 50 - 150 kg athletes) in order to maintain liver and muscle glycogen stores [1,6]. Research has also shown that athletes involved in high volume intense training (e.g., 3-6 hours per day of intense training in 1-2 workouts for 5-6 days per week) may need to consume 8-10 grams/day of carbohydrate (i.e., 400 - 1,500 grams/day for 50 - 150 kg athletes) in order to maintain muscle glycogen levels [1,6]. This would be equivalent to consuming 0.5 - 2.0 kg of spaghetti. Preferably, the majority of dietary carbohydrate should come from complex carbohydrates with a low to moderate glycemic index (e.g., whole grains, vegetables, fruit, etc). However, since it is physically difficult to consume that much carbohydrate per day when an athlete is involved in intense training, many nutritionists and the sports nutrition specialist recommend that athletes consume concentrated carbohydrate juices/drinks and/or consume high carbohydrate supplements to meet carbohydrate needs.

While consuming this amount of carbohydrate is not necessary for the fitness minded individual who only trains 3-4 times per week for 30-60 minutes, it is essential for competitive athletes engaged in intense moderate to high volume training. The general consensus in the scientific literature is the body can oxidize 1 - 1.1 gram of carbohydrate per minute or about 60 grams per hour [13]. The American College of Sports Medicine (ACSM) recommends ingesting 0.7 g/kg/hr during exercise in a 6-8% solution (i.e., 6-8 grams per 100 ml of fluid). Harger-Domitrovich et al [14] reported that 0.6 g/kg/h of maltodextrin optimized carbohydrate utilization [14]. This would be about 30 - 70 grams of CHO per hour for a 50 - 100 kg individual [15-17]. Studies also indicate that ingestion of additional amounts of carbohydrate does not further increase carbohydrate oxidation.

It should also be noted that exogenous carbohydrate oxidation rates have been shown to differ based on the type of carbohydrate consumed because they are taken up by different transporters [18-20]. For example, oxidation rates of disaccharides and polysaccharides like sucrose, maltose, and maltodextrins are high while fructose, galactose, trehalose, and isomaltulose are lower [21,22]. Ingesting combinations of glucose and sucrose or maltodextrin and fructose have been reported to promote greater exogenous carbohydrate oxidation than other forms of carbohydrate [18-26]. These studies generally indicate a ratio of 1-1.2 for maltodextrin to 0.8-1.0 fructose. For this reason, we recommend that care should be taken to consider the type of carbohydrate to ingest prior to, during, and following intense exercise in order to optimize carbohydrate availability.

### Protein

There has been considerable debate regarding protein needs of athletes [27-31]. Initially, it was recommended that athletes do not need to ingest more than the RDA for protein (i.e., 0.8 to 1.0 g/kg/d for children, adolescents and adults). However, research over the last decade has indicated that athletes engaged in intense training need to ingest about two times the RDA of protein in their diet (1.5 to 2.0 g/kg/d) in order to maintain protein balance [27,28,30,32,33]. If an insufficient amount of protein is obtained from the diet, an athlete will maintain a negative nitrogen balance, which can increase protein catabolism and slow recovery. Over time, this may lead to muscle wasting and training intolerance [1,8].

For people involved in a general fitness program, protein needs can generally be met by ingesting 0.8 - 1.0 grams/kg/day of protein. Older individuals may also benefit from a higher protein intake (e.g., 1.0 - 1.2 grams/kg/day of protein) in order to help prevent sarcopenia. It is recommended that athletes involved in moderate amounts of intense training consume 1 - 1.5 grams/kg/day of protein (50 - 225 grams/day for a 50 - 150 kg athlete) while athletes involved in high volume intense training consume 1.5 - 2.0 grams/kg/day of protein (75 - 300 grams/day for a 50 - 150 kg athlete) [34]. This protein need would be equivalent to ingesting 3 - 11 servings of chicken or fish per day for a 50 - 150 kg athlete [34]. Although smaller athletes typically can ingest this amount of protein in their normal diet, larger athletes often have difficulty consuming this much dietary protein. Additionally, a number of athletic populations have been reported to be susceptible to protein malnutrition (e.g., runners, cyclists, swimmers, triathletes, gymnasts, dancers, skaters, wrestlers, boxers, etc). Therefore, care should be taken to ensure that athletes consume a sufficient amount of quality protein in their diet in order to maintain nitrogen balance (e.g., 1.5 - 2 grams/kg/day).

However, it should be noted that not all protein is the same. Proteins differ based on the source that the protein was obtained, the amino acid profile of the protein, and the methods of processing or isolating the protein [35]. These differences influence availability of amino acids and peptides that have been reported to possess biological activity (e.g., α-lactalbumin, β-lactoglobulin, glycomacropeptides, immunoglobulins, lactoperoxidases, lactoferrin, etc). Additionally, the rate of digestion and/or absorption and metabolic activity of the protein also are important considerations [35]. For example, different types of proteins (e.g., casein and whey) are digested at different rates, which directly affect whole body catabolism and anabolism [35-38]. Therefore, care should be taken not only to make sure the athlete consumes enough protein in their diet but also that the protein is high quality. The best dietary sources of low fat, high quality protein are light skinless chicken, fish, egg white and skim milk (casein and whey) [35]. The best sources of high quality protein found in nutritional supplements are whey, colostrum, casein, milk proteins and egg protein [34,35]. Although some athletes may not need to supplement their diet with protein and some sports nutrition specialists may not think that protein supplements are necessary, it is common for a sports nutrition specialist to recommend that some athletes supplement their diet with protein in order to meet dietary protein needs and/or provide essential amino acids following exercise in order to optimize protein synthesis.

The ISSN has recently adopted a position stand on protein that highlights the following points [39]:

1. Exercising individuals need approximately 1.4 to 2.0 grams of protein per kilogram of bodyweight per day.

2. Concerns that protein intake within this range is unhealthy are unfounded in healthy, exercising individuals.

3. An attempt should be made to obtain protein requirements from whole foods, but supplemental protein is a safe and convenient method of ingesting high quality dietary protein.

4. The timing of protein intake in the time period encompassing the exercise session has several benefits including improved recovery and greater gains in fat free mass.

5. Protein residues such as branched chain amino acids have been shown to be beneficial for the exercising individual, including increasing the rates of protein synthesis, decreasing the rate of protein degradation, and possibly aiding in recovery from exercise.

6. Exercising individuals need more dietary protein than their sedentary counterparts

### Fat

The dietary recommendations of fat intake for athletes are similar to or slightly greater than those recommended for non-athletes in order to promote health. Maintenance of energy balance, replenishment of intramuscular triacylglycerol stores and adequate consumption of essential fatty acids are of greater importance among athletes and allow for somewhat increased intake [40]. This depends on the athlete's training state and goals. For example, higher-fat diets appear to maintain circulating testosterone concentrations better than low-fat diets [41-43]. This has relevance to the documented testosterone suppression which can occur during volume-type overtraining [44]. Generally, it is recommended that athletes consume a moderate amount of fat (approximately 30% of their daily caloric intake), while increases up to 50% of kcal can be safely ingested by athletes during regular high-volume training [40]. For athletes attempting to decrease body fat, however, it has been recommended that they consume 0.5 to 1 g/kg/d of fat [1]. The reason for this is that some weight loss studies indicate that people who are most successful in losing weight and maintaining the weight loss are those who ingest less than 40 g/d of fat in their diet [45,46] although this is not always the case [47]. Certainly, the type of dietary fat (e.g. n-6 versus n-3; saturation state) is a factor in such research and could play an important role in any discrepancies [48,49]. Strategies to help athletes manage dietary fat intake include teaching them which foods contain various types of fat so that they can make better food choices and how to count fat grams [1,7].

### Strategic Eating and Refueling

In addition to the general nutritional guidelines described above, research has also demonstrated that timing and composition of meals consumed may play a role in optimizing performance, training adaptations, and preventing overtraining [1,6,33,50]. In this regard, it takes about 4 hours for carbohydrate to be digested and begin being stored as muscle and liver glycogen. Consequently, pre-exercise meals should be consumed about 4 to 6 h before exercise [6]. This means that if an athlete trains in the afternoon, breakfast is the most important meal to top off muscle and liver glycogen levels. Research has also indicated that ingesting a light carbohydrate and protein snack 30 to 60 min prior to exercise (e.g., 50 g of carbohydrate and 5 to 10 g of protein) serves to increase carbohydrate availability toward the end of an intense exercise bout [51,52]. This also serves to increase availability of amino acids and decrease exercise-induced catabolism of protein [33,51,52].

When exercise lasts more than one hour, athletes should ingest glucose/electrolyte solution (GES) drinks in order to maintain blood glucose levels, help prevent dehydration, and reduce the immunosuppressive effects of intense exercise [6,53-58]. Following intense exercise, athletes should consume carbohydrate and protein (e.g., 1 g/kg of carbohydrate and 0.5 g/kg of protein) within 30 min after exercise as well as consume a high carbohydrate meal within two hours following exercise [1,31,50]. This nutritional strategy has been found to accelerate glycogen resynthesis as well as promote a more anabolic hormonal profile that may hasten recovery [59-61]. Finally, for 2 to 3 days prior to competition, athletes should taper training by 30 to 50% and consume 200 to 300 g/d of extra carbohydrate in their diet. This carbohydrate loading technique has been shown to supersaturate carbohydrate stores prior to competition and improve endurance exercise capacity [1,6,50]. Thus, the type of meal and timing of eating are important factors in maintaining carbohydrate availability during training and potentially decreasing the incidence of overtraining. The ISSN has a adopted a position stand on nutrient timing [13] that was summarized with the following points:

1. Prolonged exercise (> 60 - 90 min) of moderate to high intensity exercise will deplete the internal stores of energy, and prudent timing of nutrient delivery can help offset these changes.

2. During intense exercise, regular consumption (10 - 15 fl oz.) of a carbohydrate/electrolyte solution delivering 6 - 8% CHO (6 - 8 g CHO/100 ml fluid) should be consumed every 15 - 20 min to sustain blood glucose levels.

3. Glucose, fructose, sucrose and other high-glycemic CHO sources are easily digested, but fructose consumption should be minimized as it is absorbed at a slower rate and increases the likelihood of gastrointestinal problems.

4. The addition of PRO (0.15 - 0.25 g PRO/kg/day) to CHO at all time points, especially post-exercise, is well tolerated and may promote greater restoration of muscle glycogen when carbohydrate intakes are suboptimal.

5. Ingestion of 6 - 20 grams of essential amino acids (EAA) and 30 - 40 grams of high-glycemic CHO within three hours after an exercise bout and immediately before exercise has been shown to significantly stimulate muscle PRO synthesis.

6. Daily post-exercise ingestion of a CHO + PRO supplement promotes greater increases in strength and improvements in lean tissue and body fat % during regular resistance training.

7. Milk PRO sources (e.g. whey and casein) exhibit different kinetic digestion patterns and may subsequently differ in their support of training adaptations.

8. Addition of creatine monohydrate to a CHO + PRO supplement in conjunction with regular resistance training facilitates greater improvements in strength and body composition as compared with when no creatine is consumed.

9. Dietary focus should center on adequate availability and delivery of CHO and PRO. However, including small amounts of fat does not appear to be harmful, and may help to control glycemic responses during exercise.

10. Irrespective of timing, regular ingestion of snacks or meals providing both CHO and PRO (3:1 CHO: PRO ratio) helps to promote recovery and replenishment of muscle glycogen when lesser amounts of carbohydrate are consumed.

### Vitamins

Vitamins are essential organic compounds that serve to regulate metabolic processes, energy synthesis, neurological processes, and prevent destruction of cells. There are two primary classifications of vitamins: fat and water soluble. The fat soluble vitamins include vitamins A, D, E, & K. The body stores fat soluble vitamins and therefore excessive intake may result in toxicity. Water soluble vitamins are B vitamins and vitamin C. Since these vitamins are water soluble, excessive intake of these vitamins are eliminated in urine, with few exceptions (e.g. vitamin B6, which can cause peripheral nerve damage when consumed in excessive amounts). Table 1 describes RDA, proposed ergogenic benefit, and summary of research findings for fat and water soluble vitamins. Although research has demonstrated that specific vitamins may possess some health benefit (e.g., Vitamin E, niacin, folic acid, vitamin C, etc), few have been reported to directly provide ergogenic value for athletes. However, some vitamins may help athletes tolerate training to a greater degree by reducing oxidative damage (Vitamin E, C) and/or help to maintain a healthy immune system during heavy training (Vitamin C). Theoretically, this may help athletes tolerate heavy training leading to improved performance. The remaining vitamins reviewed appear to have little ergogenic value for athletes who consume a normal, nutrient dense diet. Since dietary analyses of athletes have found deficiencies in caloric and vitamin intake, many sports nutritionists' recommend that athletes consume a low-dose daily multivitamin and/or a vitamin enriched post-workout carbohydrate/protein supplement during periods of heavy training. An article in the Journal of the American Medical Association also recently evaluated the available medical literature and recommended that Americans consume a one-a-day low-dose multivitamin in order to promote general health. Suggestions that there is no benefit of vitamin supplementation for athletes and/or it is unethical for an sports nutrition specialist to recommend that their clients take a one-a-day multi-vitamin and/or suggest taking other vitamins that may raise HDL cholesterol levels and decrease risk of heart disease (niacin), serve as antioxidants (Vitamin E), preserve musculoskeletal function and skeletal mass (vitamin D), or may help maintain a health immune system (Vitamin C) is not consistent with current available literature.

**Table 1:** Proposed Nutritional Ergogenic Aids - Vitamins

Nutrient	RDA	Proposed Ergogenic Value	Summary of Research Findings
Vitamin A	Males 900 mcg/d Females 700 mcg/d	Constituent of rhodopsin (visual pigment) and is involved in night vision. Some suggest that vitamin A supplementation may improve sport vision.	No studies have shown that vitamin A supplementation improves exercise performance [480].

Vitamin D	5 mcg/d (age <51)	Promotes bone growth and mineralization. Enhances calcium absorption. Supplementation with calcium may help prevent bone loss in osteoperotic populations.	Co-supplementation with calcium may help prevent bone loss in athletes susceptible to osteoporosis [481]. However, vitamin D supplementation does not enhance exercise performance [480].

Vitamin E	15 mg/d	As an antioxidant, it has been shown to help prevent the formation of free radicals during intense exercise and prevent the destruction of red blood cells, improving or maintaining oxygen delivery to the muscles during exercise. Some evidence suggests that it may reduce risk to heart disease or decrease incidence of recurring heart attack.	Numerous studies show that vitamin E supplementation can decrease exercise-induced oxidative stress [482-484]. However, most studies show no effects on performance at sea level. At high altitudes, vitamin E may improve exercise performance [485]. Additional research is necessary to determine whether long-term supplementation may help athletes better tolerate training.

Vitamin K	Males 120 mcg/d Females 90 mcg/d	Important in blood clotting. There is also some evidence that it may affect bone metabolism in postmenopausal women.	Vitamin K supplementation (10 mg/d) in elite female athletes has been reported to increase calcium-binding capacity of osteocalcin and promoted a 15-20% increase in bone formation markers and a 20-25% decrease in bone resorption markers suggesting an improved balance between bone formation and resorption [486].

Thiamin (B_1_)	Males 1.2 mg/d Females 1.1 mg/d	Coenzyme (thiamin pyrophosphate) in the removal of CO_2 _from decarboxylic reactions from pyruvate to acetyl CoA and in TCA cycle. Supplementation is theorized to improve anaerobic threshold and CO_2 _transport. Deficiencies may decrease efficiency of energy systems.	Dietary availability of thiamin does not appear to affect exercise capacity when athletes have a normal intake [487].

Riboflavin (B_2_)	Males 1.3 mg/d Females 1.7 mg/d	Constituent of flavin nucleotide coenzymes involved in energy metabolism. Theorized to enhance energy availability during oxidative metabolism.	Dietary availability of riboflavin does not appear to affect exercise capacity when athletes have a normal intake [487].

Niacin (B_3_)	Males 16 mg/d Females 14 mg/d	Constituent of coenzymes involved in energy metabolism. Theorized to blunt increases in fatty acids during exercise, reduce cholesterol, enhance thermoregulation, and improve energy availability during oxidative metabolism.	Studies indicate that niacin supplementation (100-500 mg/d) can help decrease blood lipid levels and increase homocysteine levels in hypercholesteremic patients [488,489]. However, niacin supplementation (280 mg) during exercise has been reported to decrease exercise capacity by blunting the mobilization of fatty acids [490].

Pyridoxine (B_6_)	1.3 mg/d (age <51)	Has been marketed as a supplement that will improve muscle mass, strength, and aerobic power in the lactic acid and oxygen systems. It also may have a calming effect that has been linked to an improved mental strength.	In well-nourished athletes, pyridoxine failed to improve aerobic capacity, or lactic acid accumulation [487]. However, when combined with vitamins B_1 _and B_12_, it may increase serotonin levels and improve fine motor skills that may be necessary in sports like pistol shooting and archery [491,492].

Cyano-cobalamin (B_12_)	2.4 mcg/d	A coenzyme involved in the production of DNA and serotonin. DNA is important in protein and red blood cell synthesis. Theoretically, it would increase muscle mass, the oxygen-carrying capacity of blood, and decrease anxiety.	In well-nourished athletes, no ergogenic effect has been reported. However, when combined with vitamins B_1 _and B_6_, cyanocobalamin has been shown to improve performance in pistol shooting [492]. This may be due to increased levels of serotonin, a neurotransmitter in the brain, which may reduce anxiety.

Folic acid (folate)	400 mcg/d	Functions as a coenzyme in the formation of DNA and red blood cells. An increase in red blood cells could improve oxygen delivery to the muscles during exercise. Believed to be important to help prevent birth defects and may help decrease homocysteine levels.	Studies suggest that increasing dietary availability of folic acid during pregnancy can lower the incidence of birth defects [493]. Additionally, it may decrease homocysteine levels (a risk factor for heart disease) [494]. In well-nourished and folate deficient-athletes, folic acid did not improve exercise performance [495].

Pantothenic acid	5 mg/d	Acts as a coenzyme for acetyl coenzyme A (acetyl CoA). This may benefit aerobic or oxygen energy systems.	Research has reported no improvements in aerobic performance with acetyl CoA supplementation. However, one study reported a decrease in lactic acid accumulation, without an improvement in performance [496].

Beta carotene	None	Serves as an antioxidant. Theorized to help minimize exercise-induced lipid peroxidation and muscle damage.	Research indicates that beta carotene supplementation with or without other antioxidants can help decrease exercise-induced peroxidation. Over time, this may help athletes tolerate training. However, it is unclear whether antioxidant supplementation affects exercise performance [483].

Vitamin C	Males 90 mg/d Females 75 mg/d	Used in a number of different metabolic processes in the body. It is involved in the synthesis of epinephrine, iron absorption, and is an antioxidant. Theoretically, it could benefit exercise performance by improving metabolism during exercise. There is also evidence that vitamin C may enhance immunity.	In well-nourished athletes, vitamin C supplementation does not appear to improve physical performance [497,498]. However, there is some evidence that vitamin C supplementation (e.g., 500 mg/d) following intense exercise may decrease the incidence of upper respiratory tract infections [471,499,500].

Recommended Dietary Allowances (RDA) based on the 1989 Food & Nutrition Board, National Academy of Sciences-National Research Council recommendations. Updated in 2001

### Minerals

Minerals are essential inorganic elements necessary for a host of metabolic processes. Minerals serve as structure for tissue, important components of enzymes and hormones, and regulators of metabolic and neural control. Some minerals have been found to be deficient in athletes or become deficient in response to training and/or prolonged exercise. When mineral status is inadequate, exercise capacity may be reduced. Dietary supplementation of minerals in deficient athletes has generally been found to improve exercise capacity. Additionally, supplementation of specific minerals in non-deficient athletes has also been reported to affect exercise capacity. Table 2 describes minerals that have been purported to affect exercise capacity in athletes. Of the minerals reviewed, several appear to possess health and/or ergogenic value for athletes under certain conditions. For example, calcium supplementation in athletes susceptible to premature osteoporosis may help maintain bone mass. There is also recent evidence that dietary calcium may help manage body composition. Iron supplementation in athletes prone to iron deficiencies and/or anaemia has been reported to improve exercise capacity. Sodium phosphate loading has been reported to increase maximal oxygen uptake, anaerobic threshold, and improve endurance exercise capacity by 8 to 10%. Increasing dietary availability of salt (sodium chloride) during the initial days of exercise training in the heat has been reported to help maintain fluid balance and prevent dehydration. ACSM recommendations for sodium levels (340 mg) represent the amount of sodium in less than 1/8 teaspoon of salt and meet recommended guidelines for sodium ingestion during exercise (300 - 600 mg per hour or 1.7 - 2.9 grams of salt during a prolonged exercise bout) [62-65]. Finally, zinc supplementation during training has been reported to decrease exercise-induced changes in immune function. Consequently, somewhat in contrast to vitamins, there appear to be several minerals that may enhance exercise capacity and/or training adaptations for athletes under certain conditions. However, although ergogenic value has been purported for remaining minerals, there is little evidence that boron, chromium, magnesium, or vanadium affect exercise capacity or training adaptations in healthy individuals eating a normal diet. Suggestions that there is no benefit of mineral supplementation for athletes and/or it is unethical for a sports nutrition specialist to recommend that their clients take minerals for health and/or performance benefit is not consistent with current available literature.

**Table 2:** Proposed Nutritional Ergogenic Aids - Minerals

Nutrient	RDA	Proposed Ergogenic Value	Summary of Research Findings
Boron	None	Boron has been marketed to athletes as a dietary supplement that may promote muscle growth during resistance training. The rationale was primarily based on an initial report that boron supplementation (3 mg/d) significantly increased β-estradiol and testosterone levels in postmenopausal women consuming a diet low in boron.	Studies which have investigated the effects of 7 wk of boron supplementation (2.5 mg/d) during resistance training on testosterone levels, body composition, and strength have reported no ergogenic value [171,172]. There is no evidence at this time that boron supplementation during resistance-training promotes muscle growth.

Calcium	1000 mg/d (ages 19-50)	Involved in bone and tooth formation, blood clotting, and nerve transmission. Stimulates fat metabolism. Diet should contain sufficient amounts, especially in growing children/adolescents, female athletes, and postmenopausal women [174]. Vitamin D needed to assist absorption.	Calcium supplementation may be beneficial in populations susceptible to osteoporosis [501]. Additionally, calcium supplementation has been shown to promote fat metabolism and help manage body composition [292,294]. Calcium supplementation provides no ergogenic effect on exercise performance.

Chromium	Males 35 mcg/d Females 25 mcg/d (ages 19-50)	Chromium, commonly sold as chromium picolinate, has been marketed with claims that the supplement will increase lean body mass and decrease body fat levels.	Animal research indicates that chromium supplementation increases lean body mass and reduces body fat. Early research on humans reported similar results [174], however, more recent well-controlled studies reported that chromium supplementation (200 to 800 mcg/d) does not improve lean body mass or reduce body fat [176,180].

Iron	Males 8 mg/d Females 18 mg/d (age 19-50)	Iron supplements are used to increase aerobic performance in sports that use the oxygen system. Iron is a component of hemoglobin in the red blood cell, which is a carrier of oxygen.	Most research shows that iron supplements do not appear to improve aerobic performance unless the athlete is iron-depleted and/or has anemia [502].

Magnesium	Males 420 Females 320	Activates enzymes involved in protein synthesis. Involved in ATP reactions. Serum levels decrease with exercise. Some suggest that magnesium supplementation may improve energy metabolism/ATP availability.	Most well-controlled research indicates that magnesium supplementation (500 mg/d) does not affect exercise performance in athletes unless there is a deficiency [503,504].

Phosphorus (phosphate salts)	700 mg/d	Phosphate has been studied for its ability to improve all three energy systems, primarily the oxygen system or aerobic capacity.	Recent well-controlled research studies reported that sodium phosphate supplementation (4 g/d for 3 d) improved the oxygen energy system in endurance tasks [400-402]. There appears to be little ergogenic value of other forms of phosphate (i.e., calcium phosphate, potassium phosphate). More research is needed to determine the mechanism for improvement.

Potassium	2000 mg/d*	An electrolyte that helps regulate fluid balance, nerve transmission, and acid-base balance. Some suggest excessive increases or decreases in potassium may predispose athletes to cramping.	Although potassium loss during intense exercise in the heat has been anecdotally associated with muscle cramping, the etiology of cramping is unknown [505,506]. It is unclear whether potassium supplementation in athletes decreases the incidence of muscle cramping [64]. No ergogenic effects reported.

Selenium	55 mcg/d	Marketed as a supplement to increase aerobic exercise performance. Working closely with vitamin E and glutathione peroxidase (an antioxidant), selenium may destroy destructive free radical production of lipids during aerobic exercise.	Although selenium may reduce lipid peroxidation during aerobic exercise, improvements in aerobic capacity have not been demonstrated [507,508].

Sodium	500 mg/d*	An electrolyte that helps regulate fluid balance, nerve transmission, and acid-base balance. Excessive decreases in sodium may predispose athletes to cramping and hyponatremia.	During the first several days of intense training in the heat, a greater amount of sodium is lost in sweat. Additionally, prolonged ultraendurance exercise may decrease sodium levels leading to hyponatremia. Increasing salt availability during heavy training in the heat has been shown to help maintain fluid balance and prevent hyponatremia [64,509].

Vanadyl sulfate (vanadium)	None	Vanadium may be involved in reactions in the body that produce insulin-like effects on protein and glucose metabolism. Due to the anabolic nature of insulin, this has brought attention to vanadium as a supplement to increase muscle mass, enhance strength and power.	Limited research has shown that type 2 diabetics may improve their glucose control; however, there is no proof that vanadyl sulfate has any effect on muscle mass, strength, or power [248,249].

Zinc	Males 11 mg/d Females 8 mg/d	Constituent of enzymes involved in digestion. Associated with immunity. Theorized to reduce incidence of upper respiratory tract infections in athletes involved in heavy training.	Studies indicate that zinc supplementation (25 mg/d) during training minimized exercise-induced changes in immune function [55,473,510,511].

Recommended Dietary Allowances (RDA) based on the 2002 Food & Nutrition Board, National Academy of Sciences-National Research Council recommendations.

* Estimated minimum requirement

### Water

The most important nutritional ergogenic aid for athletes is water. Exercise performance can be significantly impaired when 2% or more of body weight is lost through sweat. For example, when a 70-kg athlete loses more than 1.4 kg of body weight during exercise (2%), performance capacity is often significantly decreased. Further, weight loss of more than 4% of body weight during exercise may lead to heat illness, heat exhaustion, heat stroke, and possibly death [58]. For this reason, it is critical that athletes consume a sufficient amount of water and/or GES sports drinks during exercise in order to maintain hydration status. The normal sweat rate of athletes ranges from 0.5 to 2.0 L/h depending on temperature, humidity, exercise intensity, and their sweat response to exercise [58]. This means that in order to maintain fluid balance and prevent dehydration, athletes need to ingest 0.5 to 2 L/h of fluid in order to offset weight loss. This requires frequent ingestion of 6-8 oz of cold water or a GES sports drink every 5 to 15-min during exercise [58,66-69]. Athletes and should not depend on thirst to prompt them to drink because people do not typically get thirsty until they have lost a significant amount of fluid through sweat. Additionally, athletes should weigh themselves prior to and following exercise training to ensure that they maintain proper hydration [58,66-69]. The athlete should consume 3 cups of water for every pound lost during exercise in order adequately rehydrate themselves [58]. Athletes should train themselves to tolerate drinking greater amounts of water during training and make sure that they consume more fluid in hotter/humid environments. Preventing dehydration during exercise is one of the most effective ways to maintain exercise capacity. Finally, inappropriate and excessive weight loss techniques (e.g., cutting weight in saunas, wearing rubber suits, severe dieting, vomiting, using diuretics, etc) are extremely dangerous and should be prohibited. Sports nutrition specialists can play an important role in educating athletes and coaches about proper hydration methods and supervising fluid intake during training and competition.

## Dietary Supplements and Athletes

Most of the work we do with athletes regarding sports nutrition is to teach them and their coaches how to structure their diet and time food intake to optimize performance and recovery. Dietary supplements can play a meaningful role in helping athletes consume the proper amount of calories, carbohydrate, and protein in their diet. However, they should be viewed as supplements to the diet, not replacements for a good diet. While it is true that most dietary supplements available for athletes have little scientific data supporting their potential role to enhance training and/or performance, it is also true that a number of nutrients and/or dietary supplements have been shown to help improve performance and/or recovery. Supplementation with these nutrients can help augment the normal diet to help optimize performance. Sports nutrition specialists must be aware of the current data regarding nutrition, exercise, and performance and be honest about educating their clients about results of various studies (whether pro or con). With the proliferation of information available about nutritional supplements to the consumer, the sports nutrition specialist, nutritionist, and nutrition industry lose credibility when they do not accurately describe results of various studies to the public. The following outlines several classifications of nutritional supplements that are often taken by athletes and categorizes them into 'apparently effective', 'possibly effective', 'too early to tell', and 'apparently ineffective' supplements based on interpretation of the literature. It should be noted that this analysis focuses primarily on whether the proposed nutrient has been found to affect exercise and/or training adaptations based on the current available literature. Additional research may or may not reveal ergogenic value, possibly altering its classification. It should be also noted that although there may be little ergogenic value to some nutrients, there may be some potential health benefits that may be helpful for some populations. Therefore, just because a nutrient does not appear to affect performance and/or training adaptations, that does not mean it does not have possible health benefits for athletes.

### Convenience Supplements

Convenience supplements are meal replacement powders (MRP's), ready to drink supplements (RTD's), energy bars, and energy gels. They currently represent the largest segment of the dietary supplement industry representing 50 - 75% of most company's sales. They are typically fortified with vitamins and minerals and differ on the amount of carbohydrate, protein, and/or fat they contain. They may also vary based whether they are fortified with various nutrients purported to promote weight gain, enhance weight loss, and/or improve performance. Most people view these supplements as a nutrient dense snack and/or use them to help control caloric intake when trying to gain and/or lose weight. In our view, MRP's, RTD's, and energy bars/gels can provide a convenient way for people to meet specific dietary needs and/or serve as good alternatives to fast food other foods of lower nutritional value. Use of these types of products can be particularly helpful in providing carbohydrate, protein, and other nutrients prior to and/or following exercise in an attempt to optimize nutrient intake when an athlete doesn't have time to sit down for a good meal or wants to minimize food volume. However, they should be used to improve dietary availability of macronutrients - not as a replacement for a good diet. Care should also be taken to make sure they do not contain any banned or prohibited nutrients.

### Muscle Building Supplements

The following provides an analysis of the literature regarding purported weight gain supplements and our general interpretation of how they should be categorized based on this information. Table 3 summarizes how we currently classify the ergogenic value of a number of purported performance-enhancing, muscle building, and fat loss supplements based on an analysis of the available scientific evidence.

**Table 3:** Summary of categorization of dietary supplements based on available literature.

Category	Muscle Building Supplements	Weight Loss Supplements	Performance Enhancement
Apparently effective and generally safe	Weight gain powders Creatine Protein EAA	Low-calorie foods, MRPs, and RTDs Ephedra, caffeine, and salicin-containing thermogenic supplements taken at recommended doses in appropriate populations (ephedra banned by FDA)	Water and sports drinks Carbohydrate Creatine Sodium phosphate Sodium bicarbonate Caffeine B-alanine

Possibly effective	HMB (untrained individuals initiating training) BCAA	High-fiber diets Calcium Green tea extract Conjugated Linoleic Acids	Post-exercise carbohydrate & protein EAA BCAA HMB Glycerol

Too early to tell	α-Ketoglutarate α-Ketoisocaproate Ecdysterones Growth hormone releasing peptides and secretogues Ornithine α-Ketoglutarate Zinc/magnesium aspartate	Gymnema sylvestre, chitosan) Phosphatidl Choline Betaine Coleus Forskolin DHEA Psychotropic Nutrients/Herbs	Medium chain triglycerides

Apparently not effective and/or dangerous	Glutamine Smilax Isoflavones Sulfo-polysaccharides (myostatin inhibitors) Boron Chromium Conjugated linoleic acids Gamma oryzanol Prohormones Tribulus terrestris Vanadyl sulfate (vanadium)	Calcium Pyruvate Chitosan Chromium (non-diabetics) HCA L-Carnitine Phosphates Herbal diuretics	Glutamine Ribose Inosine

#### Apparently Effective

##### Weight Gain Powders

One of the most common means athletes have employed to increase muscle mass is to add extra calories to the diet. Most athletes "bulk up" in this manner by consuming extra food and/or weight gain powders. In order to increase skeletal muscle mass, there must be adequate energy intake (anabolic reactions are endergonic and therefore require adequate energy intake). Studies have consistently shown that simply adding an extra 500 - 1,000 calories per day to your diet in conjunction with resistance training will promote weight gain [31,33]. However, only about 30 - 50% of the weight gained on high calorie diets is muscle while the remaining amount of weight gained is fat. Consequently, increasing muscle mass by ingesting a high calorie diet can help build muscle but the accompanying increase in body fat may not be desirable for everyone. Therefore, we typically do not recommend this type of weight gain approach [39].

##### Creatine monohydrate

In our view, the most effective nutritional supplement available to athletes to increase high intensity exercise capacity and muscle mass during training is creatine monohydrate. Numerous studies have indicated that creatine supplementation increases body mass and/or muscle mass during training [70] Gains are typically 2 - 5 pounds greater than controls during 4 - 12 weeks of training [71]. The gains in muscle mass appear to be a result of an improved ability to perform high intensity exercise enabling an athlete to train harder and thereby promote greater training adaptations and muscle hypertrophy [72-75]. The only clinically significant side effect occasionally reported from creatine monohydrate supplementation has been the potential for weight gain [71,76-78] Although concerns have been raised about the safety and possible side effects of creatine supplementation [79,80], recent long-term safety studies have reported no apparent side effects [78,81,82] and/or that creatine monohydrate may lessen the incidence of injury during training [83-85]. Additionally a recent review was published which addresses some of the concerns and myths surrounding creatine monohydrate supplementation [86]. Consequently, supplementing the diet with creatine monohydrate and/or creatine containing formulations seems to be a safe and effective method to increase muscle mass. The ISSN position stand on creatine monohydrate [87] summarizes their findings as this:

1. Creatine monohydrate is the most effective ergogenic nutritional supplement currently available to athletes in terms of increasing high-intensity exercise capacity and lean body mass during training.

2. Creatine monohydrate supplementation is not only safe, but possibly beneficial in regard to preventing injury and/or management of select medical conditions when taken within recommended guidelines.

3. There is no compelling scientific evidence that the short- or long-term use of creatine monohydrate has any detrimental effects on otherwise healthy individuals.

4. If proper precautions and supervision are provided, supplementation in young athletes is acceptable and may provide a nutritional alternative to potentially dangerous anabolic drugs.

5. At present, creatine monohydrate is the most extensively studied and clinically effective form of creatine for use in nutritional supplements in terms of muscle uptake and ability to increase high-intensity exercise capacity.

6. The addition of carbohydrate or carbohydrate and protein to a creatine supplement appears to increase muscular retention of creatine, although the effect on performance measures may not be greater than using creatine monohydrate alone.

7. The quickest method of increasing muscle creatine stores appears to be to consume ~0.3 grams/kg/day of creatine monohydrate for at least 3 days followed by 3-5 g/d thereafter to maintain elevated stores. Ingesting smaller amounts of creatine monohydrate (e.g., 2-3 g/d) will increase muscle creatine stores over a 3-4 week period, however, the performance effects of this method of supplementation are less supported.

8. Creatine monohydrate has been reported to have a number of potentially beneficial uses in several clinical populations, and further research is warranted in these areas.

##### Protein

As previously described, research has indicated that people undergoing intense training may need additional protein in their diet to meet protein needs (i.e., 1.4 - 2.0 grams/day [13,39]. People who do not ingest enough protein in their diet may exhibit slower recovery and training adaptations [33]. Protein supplements offer a convenient way to ensure that athletes consume quality protein in the diet and meet their protein needs. However, ingesting additional protein beyond that necessary to meet protein needs does not appear to promote additional gains in strength and muscle mass. The research focus over recent years has been to determine whether different types of protein (e.g., whey, casein, soy, milk proteins, colostrum, etc) and/or various biologically active protein subtypes and peptides (e.g., α-lactalbumin, β-lactoglobulin, glycomacropeptides, immunoglobulins, lactoperoxidases, lactoferrin, etc) have varying effects on the physiological, hormonal, and/or immunological responses to training [88-91]. In addition, a significant amount of research has examined whether timing of protein intake and/or provision of specific amino acids may play a role in protein synthesis and/or training adaptations, conducted mostly in untrained populations [92-105]. Although more research is necessary in this area, evidence clearly indicates that protein needs of individuals engaged in intense training are elevated, different types of protein have varying effects on anabolism and catabolism, that different types of protein subtypes and peptides have unique physiological effects, and timing of protein intake may play an important role in optimizing protein synthesis following exercise. Therefore, it is simplistic and misleading to suggest that there is no data supporting contentions that athletes need more protein in their diet and/or there is no potential ergogenic value of incorporating different types of protein into the diet. It is the position stand of ISSN that exercising individuals need approximately 1.4 to 2.0 grams of protein per kilogram of bodyweight per day. This is greater than the RDA recommendations for sedentary individuals. According to the current literature we know that the addition of protein and or BCAA before or after resistance training can increase protein synthesis and gains in lean mass beyond normal adaptation. However, it should be noted that gains have primarily been observed in untrained populations unless the supplement contained other nutrients like creatine monohydrate [13,39].

##### Essential Amino Acids (EAA)

Recent studies have indicated that ingesting 3 to 6 g of EAA prior to [105,106] and/or following exercise stimulates protein synthesis [92,93,98-101,105]. Theoretically, this may enhance gains in muscle mass during training. To support this theory, a study by Esmarck and colleagues [107] found that ingesting EAA with carbohydrate immediately following resistance exercise promoted significantly greater training adaptations in elderly, untrained men, as compared to waiting until 2-hours after exercise to consume the supplement. Although more data is needed, there appears to be strong theoretical rationale and some supportive evidence that EAA supplementation may enhance protein synthesis and training adaptations. Because EAA's include BCAA's, it is probable that positive effects on protein synthesis from EAA ingestion are likely due to the BCAA content [108,109]. Garlick and Grant [109] infused glucose into growing rats to achieve a concentration of insulin secretion that was insufficient to stimulate protein synthesis by itself. In addition to this, all eight essential amino acids with glucose was infused into another group and then in a third group the investigators only infused the BCAA's along with the glucose. Compared with the glucose infusion alone, protein synthesis was stimulated equally by the essential amino acids and the BCAAs. This demonstrates that the BCAAs are the key amino acids that stimulate protein synthesis. The ISSN position stand on protein concluded that BCAAs have been shown to acutely stimulate protein synthesis, aid in glycogen resynthesis, delaying the onset of fatigue, and help maintain mental function in aerobic-based exercise. It was concluded that consuming BCAAs (in addition to carbohydrates) before, during, and following an exercise bout would be recommended safe and effective [39].

#### Possibly Effective

##### β-hydroxy β-methylbutyrate (HMB)

HMB is a metabolite of the amino acid leucine. Leucine and metabolites of leucine have been reported to inhibit protein degradation [110]. Supplementing the diet with 1.5 to 3 g/d of calcium HMB during training has been typically reported to increase muscle mass and strength particularly among untrained subjects initiating training [111-116] and the elderly [117]. Gains in muscle mass are typically 0.5 to 1 kg greater than controls during 3 - 6 weeks of training. There is also evidence that HMB may lessen the catabolic effects of prolonged exercise [118,119] and that there may be additive effects of co-ingesting HMB with creatine [120,121]. However, the effects of HMB supplementation in athletes are less clear. Most studies conducted on trained subjects have reported non-significant gains in muscle mass possibly due to a greater variability in response of HMB supplementation among athletes [122-124]. Consequently, there is fairly good evidence showing that HMB may enhance training adaptations in individuals initiating training. However, additional research is necessary to determine whether HMB may enhance training adaptations in trained athletes.

##### Branched Chain Amino Acids (BCAA)

BCAA supplementation has been reported to decrease exercise-induced protein degradation and/or muscle enzyme release (an indicator of muscle damage) possibly by promoting an anti-catabolic hormonal profile [31,51,125]. Theoretically, BCAA supplementation during intense training may help minimize protein degradation and thereby lead to greater gains in fat-free mass. There is some evidence to support this hypothesis. For example, Schena and colleagues [126] reported that BCAA supplementation (~10 g/d) during 21-days of trekking at altitude increased fat free mass (1.5%) while subjects ingesting a placebo had no change in muscle mass. Bigard and associates [127] reported that BCAA supplementation appeared to minimize loss of muscle mass in subjects training at altitude for 6-weeks. Finally, Candeloro and coworkers [128] reported that 30 days of BCAA supplementation (14 grams/day) promoted a significant increase in muscle mass (1.3%) and grip strength (+8.1%) in untrained subjects. A recent published abstract [129] reported that resistance trained subjects ingesting 14 grams of BCAA during 8 weeks of resistance training experienced a significantly greater gain in body weight and lean mass as compared to a whey protein supplemented group and a carbohydrate placebo group. Specifically, the BCAA group gained 2 kg of body mass and 4 kg of lean body mass. In contrast, the whey protein and carbohydrate groups both gained an additional 1 kg of body mass and 2 kg and 1 kg of lean body mass, respectively. It cannot be overstated that this investigation was published as an abstract, was conducted in a gym setting, and has not undergone the rigors of peer review at this time. Although more research is necessary, these findings suggest that BCAA supplementation may have some impact on body composition.

#### Too Early to Tell

##### α-ketoglutarate (α-KG)

α-KG is an intermediate in the Krebs cycle that is involved in aerobic energy metabolism. There is some clinical evidence that α-KG may serve as an anticatabolic nutrient after surgery [130,131]. However, it is unclear whether α-KG supplementation during training may affect training adaptations.

##### α-Ketoisocaproate (KIC)

KIC is a branched-chain keto acid that is a metabolite of leucine metabolism. In a similar manner as HMB, leucine and metabolites of leucine are believed to possess anticatabolic properties [132]. There is some clinical evidence that KIC may spare protein degradation in clinical populations [133,134]. Theoretically, KIC may help minimize protein degradation during training possibly leading to greater training adaptations. However, we are not aware of any studies that have evaluated the effects of KIC supplementation during training on body composition.

##### Ecdysterones

Ecdysterones (also known as ectysterone, 20 Beta-Hydroxyecdysterone, turkesterone, ponasterone, ecdysone, or ecdystene) are naturally derived phytoecdysteroids (i.e., insect hormones). They are typically extracted from the herbs *Leuza rhaptonticum *sp., *Rhaponticum carthamoides*, or *Cyanotis vaga*. They can also be found in high concentrations in the herb Suma (also known as Brazilian Ginseng or Pfaffia). Research from Russia and Czechoslovakia conducted over the last 30 years indicates that ecdysterones may possess some potentially beneficial physiological effects in insects and animals [135-140]. However, since most of the data on ecdysterones have been published in obscure journals, results are difficult to interpret. Wilborn and coworkers [141] gave resistance trained males 200 mg of 20-hydroxyecdysone per day during 8-weeks of resistance training. It was reported that the 20-hydroxyecdysone supplementation had no effect on fat free mass or anabolic/catabolic hormone status [141]. Due to the findings of this well controlled study in humans, ecdysterone supplementation at a dosage of 200 mg per day appears to be ineffective in terms of improving lean muscle mass. While future studies may find some ergogenic value of ecdysterones, it is our view that it is too early to tell whether phytoecdysteroids serve as a safe and effective nutritional supplement for athletes.

##### Growth Hormone Releasing Peptides (GHRP) and Secretagogues

Research has indicated that growth hormone releasing peptides (GHRP) and other non-peptide compounds (secretagogues) appear to help regulate growth hormone (GH) release [142,143]. These observations have served as the basis for development of nutritionally-based GH stimulators (e.g., amino acids, pituitary peptides, "pituitary substances", *Macuna pruriens*, broad bean, alpha-GPC, etc). Although there is clinical evidence that pharmaceutical grade GHRP's and some non-peptide secretagogues can increase GH and IGF-1 levels at rest and in response to exercise, it has not been demonstrated that such increases lead to an increase in skeletal muscle mass [144].

##### Ornithine-α-ketoglutarate (OKG)

OKG (via enteral feeding) has been shown to significantly shorten wound healing time and improve nitrogen balance in severe burn patients [145,146]. Because of its ability to improve nitrogen balance, OKG may provide some value for athletes engaged in intense training. A study by Chetlin and colleagues [147] reported that OKG supplementation (10 grams/day) during 6-weeks of resistance training promoted greater gains in bench press. However, no significant differences were observed in squat strength, training volume, gains in muscle mass, or fasting insulin and growth hormone. Therefore, additional research is needed before conclusions can be drawn.

##### Zinc/Magnesium Aspartate (ZMA)

The main ingredients in ZMA formulations are zinc monomethionine aspartate, magnesium aspartate, and vitamin B-6. The rationale of ZMA supplementation is based on studies suggesting that zinc and magnesium deficiency may reduce the production of testosterone and insulin like growth factor (IGF-1). ZMA supplementation has been theorized to increase testosterone and IGF-1 leading to greater recovery, anabolism, and strength during training. In support of this theory, Brilla and Conte [148] reported that a zinc-magnesium formulation increased testosterone and IGF-1 (two anabolic hormones) leading to greater gains in strength in football players participating in spring training. In another study conducted by Wilborn et al. [149], resistance trained males ingested a ZMA supplement and found no such increases in either total or free testosterone. In addition, this investigation also assessed changes in fat free mass and no significant differences were observed in relation to fat free mass in those subjects taking ZMA. The discrepancies concerning the two aforementioned studies may be explained by deficiencies of these minerals. Due to the role that zinc deficiency plays relative to androgen metabolism and interaction with steroid receptors [150], when there are deficiencies of this mineral, testosterone production may suffer. In the study showing increases in testosterone levels [148], there were depletions of zinc and magnesium in the placebo group over the duration of the study. Hence, increases in testosterone levels could have been attributed to impaired nutritional status rather than a pharmacologic effect. More research is needed to further evaluate the role of ZMA on body composition and strength during training before definitive conclusions can be drawn.

#### Apparently Ineffective

##### Glutamine

Glutamine is the most plentiful non-essential amino acid in the body and plays a number of important physiological roles [31,108,109] Glutamine has been reported to increase cell volume and stimulate protein [151,152] and glycogen synthesis [153]. Despite its important role in physiological roles, there is no compelling evidence to support glutamine supplementation in terms of increasing lean body mass. One study that is often cited in support of glutamine supplementation and its role in increasing muscle mass was published by Colker and associates [154]. It was reported that subjects who supplemented their diet with glutamine (5 grams) and BCAA (3 grams) enriched whey protein during training promoted about a 2 pound greater gain in muscle mass and greater gains in strength than ingesting whey protein alone. While a 2 pound increase in lean body mass was observed, it is likely that these gains were due to the BCAAs that were added to the whey protein. In a well-designed investigation, Candow and co-workers [155] studied the effects of oral glutamine supplementation combined with resistance training in young adults. Thirty-one participants were randomly allocated to receive either glutamine (0.9 g/kg of lean tissue mass) or a maltodextrin placebo (0.9 g/kg of lean tissue mass) during 6 weeks of total body resistance training. At the end of the 6-week intervention, the authors concluded glutamine supplementation during resistance training had no significant effect on muscle performance, body composition or muscle protein degradation in young healthy adults. While there may be other beneficial uses for glutamine supplementation, there does not appear to be any scientific evidence that it supports increases in lean body mass or muscular performance.

##### Smilax officinalis (SO)

SO is a plant that contains plant sterols purported to enhance immunity as well as provide an androgenic effect on muscle growth [1]. Some data supports the potential immune enhancing effects of SO. However, we are not aware of any data that show that SO supplementation increases muscle mass during training.

##### Isoflavones

Isoflavones are naturally occurring non-steroidal phytoestrogens that have a similar chemical structure as ipriflavone (a synthetic flavonoid drug used in the treatment of osteoporosis) [156-158]. For this reason, soy protein (which is an excellent source of isoflavones) and isoflavone extracts have been investigated in the possible treatment of osteoporosis. Results of these studies have shown promise in preventing declines in bone mass in post-menopausal women as well as reducing risks to side effects associated with estrogen replacement therapy. More recently, the isoflavone extracts 7-isopropoxyisoflavone (ipriflavone) and 5-methyl-7-methoxy-isoflavone (methoxyisoflavone) have been marketed as "powerful anabolic" substances. These claims have been based on research described in patents filed in Hungary in the early 1970s [159,160]. Aubertin-Leheudre M, et al. [161] investigated the effects that isoflavone supplementation would have on fat-free mass in obese, sarcopenic postmenopausal women. Eighteen sarcopenic-obese women ingested 70 mg of isoflavones per day (44 mg of daidzein, 16 mg glycitein and 10 mg genistein) or a placebo for six months. There was no exercise intervention in the investigation, only the isoflavone supplementation. At the end of the six month intervention, it was reported that there was no difference in total body fat free mass between the isoflavone and placebo groups, but there was a significant increase in the appendicular (arms and legs) fat free mass in the isoflavone supplemented group but not the placebo group. Findings from this study have some applications to sedentary, postmenopausal women. However, there are currently no peer-reviewed data indicating that isoflavone supplementation affects exercise, body composition, or training adaptations in physically active individuals.

##### Sulfo-Polysaccharides (Myostatin Inhibitors)

Myostatin or growth differentiation factor 8 (GDF-8) is a transforming growth factor that has been shown to serve as a genetic determinant of the upper limit of muscle size and growth [162]. Recent research has indicated that eliminating and/or inhibiting myostatin gene expression in mice [163] and cattle [164-166] promotes marked increases in muscle mass during early growth and development. The result is that these animals experience what has been termed as a "double-muscle" phenomenon apparently by allowing muscle to grow beyond its normal genetic limit. In agriculture research, eliminating and/or inhibiting myostatin may serve as an effective way to optimize animal growth leading to larger, leaner, and a more profitable livestock yield. In humans, inhibiting myostatin gene expression has been theorized as a way to prevent or slow down muscle wasting in various diseases, speed up recovery of injured muscles, and/or promote increases in muscle mass and strength in athletes [167]. While these theoretical possibilities may have great promise, research on the role of myostatin inhibition on muscle growth and repair is in the very early stages - particularly in humans. There is some evidence that myostatin levels are higher in the blood of HIV positive patients who experience muscle wasting and that myostatin levels negatively correlate with muscle mass [162]. There is also evidence that myostatin gene expression may be fiber specific and that myostatin levels may be influenced by immobilization in animals [168]. Additionally, a study by Ivey and colleagues [167] reported that female athletes with a less common myostatin allele (a genetic subtype that may be more resistant to myostatin) experienced greater gains in muscle mass during training and less loss of muscle mass during detraining. No such pattern was observed in men with varying amounts of training histories and muscle mass. These early studies suggest that myostatin may play a role in regulating muscle growth to some degree. Some nutrition supplement companies have marketed sulfo-polysaccharides (derived from a sea algae called *Cytoseira canariensis*) as a way to partially bind the myostatin protein in serum. When untrained males supplemented with 1200 mg/day of *Cystoseira canariensis *in conjunction with a twelve week resistance training regimen, it was reported that there were no differences between the supplemented group and the placebo group in relation to fat-free mass, muscle strength, thigh volume/mass, and serum myostatin [169]. Interestingly, a recent paper by Seremi and colleagues [170] reported that resistance training reduced serum myostatin levels and that creatine supplementation in conjunction with resistance training promoted further reductions. Nevertheless, though the research is limited, there is currently no published data supporting the use of sulfo-polysaccharides as a muscle building supplement.

##### Boron

Boron is a trace mineral proposed to increase testosterone levels and promote anabolism. Several studies have evaluated the effects of boron supplementation during training on strength and body composition alterations. These studies (conducted on male bodybuilders) indicate that boron supplementation (2.5 mg/d) appears to have no impact on muscle mass or strength [171,172].

##### Chromium

Chromium is a trace mineral that is involved in carbohydrate and fat metabolism. Clinical studies have suggested that chromium may enhance the effects of insulin particularly in diabetic populations. Since insulin is an anti-catabolic hormone and has been reported to affect protein synthesis, chromium supplementation has been theorized to serve as an anabolic nutrient. Theoretically, this may increase anabolic responses to exercise. Although some initial studies reported that chromium supplementation increased gains in muscle mass and strength during training particularly in women [173-175], most well-controlled studies [176] that have been conducted since then have reported no benefit in healthy individuals taking chromium (200-800 mcg/d) for 4 to 16-weeks during training [177-183]. Consequently, it appears that although chromium supplementation may have some therapeutic benefits for diabetics, chromium does not appear to be a muscle-building nutrient for athletes.

##### Conjugated Linoleic Acids (CLA)

Animal studies indicate that adding CLA to dietary feed decreases body fat, increases muscle and bone mass, has anti-cancer properties, enhances immunity, and inhibits progression of heart disease [184-186]. Consequently, CLA supplementation in humans has been suggested to help manage body composition, delay loss of bone, and provide health benefit. Although animal studies are impressive [187-189] and some studies suggests benefit over time at some but not all dosages [190-192], there is little current evidence that CLA supplementation during training can affect lean tissue accretion [193,194]. As will be discussed below, there appears to be more promise of CLA as a supplement to promote general health and/or reductions in fat mass over time.

##### Gamma Oryzanol (Ferulic Acid)

Gamma oryzanol is a plant sterol theorized to increase anabolic hormonal responses during training [195]. Although data are limited, one study reported no effect of 0.5 g/d of gamma oryzanol supplementation on strength, muscle mass, or anabolic hormonal profiles during 9-weeks of training [196].

##### Prohormones

Testosterone and growth hormone are two primary hormones in the body that serve to promote gains in muscle mass (i.e., anabolism) and strength while decreasing muscle breakdown (catabolism) and fat mass [197-204]. Testosterone also promotes male sex characteristics (e.g., hair, deep voice, etc) [198]. Low level anabolic steroids are often prescribed by physicians to prevent loss of muscle mass for people with various diseases and illnesses [205-216]. It is well known that athletes have experimented with large doses of anabolic steroids in an attempt to enhance training adaptations, increase muscle mass, and/or promote recovery during intense training [198-200,203,204,217]. Research has generally shown that use of anabolic steroids and growth hormone during training can promote gains in strength and muscle mass [197,202,204,210,213,218-225]. However, a number of potentially life threatening adverse effects of steroid abuse have been reported including liver and hormonal dysfunction, hyperlipidemia (high cholesterol), increased risk to cardiovascular disease, and behavioral changes (i.e., steroid rage) [220,226-230]. Some of the adverse effects associated with the use of these agents are irreversible, particularly in women [227]. For this reason, anabolic steroids have has been banned by most sport organizations and should be avoided unless prescribed by a physician to treat an illness.

Prohormones (androstenedione, 4-androstenediol, 19-nor-4-androstenedione, 19-nor-4-androstenediol, 7-keto DHEA, and DHEA, etc) are naturally derived precursors to testosterone or other anabolic steroids. Prohormones have become popular among body builders because they believe they are natural boosters of anabolic hormones. Consequently, a number of over-the-counter supplements contain prohormones. While there is some data indicating that prohormones increase testosterone levels [231,232], there is virtually no evidence that these compounds affect training adaptations in younger men with normal hormone levels. In fact, most studies indicate that they do not affect testosterone and that some may actually increase estrogen levels and reduce HDL-cholesterol [220,231,233-238]. Consequently, although there may be some potential applications for older individuals to replace diminishing androgen levels, it appears that prohormones have no training value. Since prohormones are "steroid-like compounds", most athletic organizations have banned their use. Use of nutritional supplements containing prohormones will result in a positive drug test for anabolic steroids. Use of supplements knowingly or unknowingly containing prohormones have been believed to have contributed to a number of recent positive drug tests among athletes. Consequently, care should be taken to make sure that any supplement an athlete considers taking does not contain prohormone precursors particularly if their sport bans and tests for use of such compounds. It is noteworthy to mention that many prohormones are not lawful for sale in the USA since the passage of the Anabolic Steroid Control Act of 2004. The distinctive exception to this is DHEA, which has been the subject of numerous clinical studies in aging populations.

Rather than provide the body with a precursor to testosterone, a more recent technique to enhance endogenous testosterone has been to inhibit aromatase activity [239]. Two studies have investigated the effects of aromatase inhibitors (androst-4-ene-3,6,17-trione) [240] and (hydroxyandrost-4-ene-6,17-dioxo-3-THP ether and 3,17-diketo-androst-1,4,6-triene) [241]. In both of these investigations, it was reported that free testosterone and dihydrotesterone levels were significantly increased. Muscle mass/fat free mass was not measured in one investigation [240] and no changes were observed in fat free mass in the other investigation [241].

##### Tribulus terrestris

*Tribulus terrestris *(also known as puncture weed/vine or caltrops) is a plant extract that has been suggested to stimulate leutinizing hormone (LH) which stimulates the natural production of testosterone [132]. Consequently, Tribulus has been marketed as a supplement that can increase testosterone and promote greater gains in strength and muscle mass during training. Several recent studies have indicated that Tribulus supplementation appears to have no effects on body composition or strength during training [242-244].

##### Vanadyl Sulfate (Vanadium)

In a similar manner as chromium, vanadyl sulfate is a trace mineral that has been found to affect insulin-sensitivity and may affect protein and glucose metabolism [132,245]. For this reason, vanadyl sulfate has been purported to increase muscle mass and strength during training. Although there may be some clinical benefits for diabetics (with a therapeutic dose of at least 50 mg vanadyl sulfate twice daily [246,247], vanadyl sulfate supplementation does not appear to have any effect on strength or muscle mass during training in non-diabetic, weight training individuals [248,249].

### Weight Loss Supplements

Although exercise and proper diet remain the best way to promote weight loss and/or manage body composition, a number of nutritional approaches have been investigated as possible weight loss methods (with or without exercise). The following overviews the major types of weight loss products available and discusses whether any available research supports their use. See Table 3 for a summary.

#### Apparently Effective

##### Low Calorie Diet Foods & Supplements

Most of the products in this category represent low fat/carbohydrate, high protein food alternatives [250]. They typically consist of pre-packaged food, bars, MRP, or RTD supplements. They are designed to provide convenient foods/snacks to help people follow a particular low calorie diet plan. In the scientific literature, diets that provide less than 1000 calories per day are known as very low calorie diets (VLCD's). Pre-packaged food, MRP's, and/or RTD's are often provided in VLCD plans to help people cut calories. In most cases, VLCD plans recommend behavioural modification and that people start a general exercise program.

Research on the safety and efficacy of people maintaining VLCD's generally indicate that they can promote weight loss. For example, Hoie et al [251] reported that maintaining a VLCD for 8-weeks promoted a 27 lbs (12.6%) loss in total body mass, a 21 lbs loss in body fat (23.8%), and a 7 lbs (5.2%) loss in lean body mass in 127 overweight volunteers. Bryner and colleagues [252] reported that addition of a resistance training program while maintaining a VLCD (800 kcal/d for 12-weeks) resulted in a better preservation of lean body mass and resting metabolic rate compared to subjects maintaining a VLCD while engaged in an endurance training program. Meckling and Sherfey [253] reported that the combination of high protein and exercise was the most effective intervention for weight loss and was superior to a low-fat, high-carbohydrate diet in promoting weight loss and nitrogen balance regardless of the presence of an exercise intervention. Recent studies indicate that high protein/low fat VLCD's may be better than high carbohydrate/low fat diets in promoting weight loss [46,253-260]. The reason for this is that typically when people lose weight about 40-50% of the weight loss is muscle which decreases resting energy expenditure. Increasing protein intake during weight loss helps preserve muscle mass and resting energy expenditure to a better degree than high carbohydrate diets [261,262]. These findings and others indicate that VLCD's (typically using MRP's and/or RTD's as a means to control caloric intake) can be effective particularly as part of an exercise and behavioural modification program. Most people appear to maintain at least half of the initial weight lost for 1-2 years but tend to regain most of the weight back within 2-5 years. Therefore, although these diets may help people lose weight on the short-term, it is essential people who use them follow good diet and exercise practices in order to maintain the weight loss. The addition of dietary protein whether in whole food form or meal replacement form could assist in this weight maintenance due to the fact that the retention of muscle mass is greater than in high carbohydrate/low-fat weight loss trials?

##### Ephedra, Caffeine, and Silicin

Thermogenics are supplements designed to stimulate metabolism thereby increasing energy expenditure and promote weight loss. They typically contain the "ECA" stack of ephedra alkaloids (e.g., Ma Haung, 1R,2S Nor-ephedrine HCl, Sida Cordifolia), caffeine (e.g., Gaurana, Bissey Nut, Kola) and aspirin/salicin (e.g., Willow Bark Extract). The first of the three traditional thermogenics is now banned by the FDA however the safety associated with the ingestion of ephedra is debated. More recently, other potentially thermogenic nutrients have been added to various thermogenic formulations. For example, thermogenic supplements may also contain synephrine (e.g., Citrus Aurantum, Bitter Orange), calcium & sodium phosphate, thyroid stimulators (e.g., guggulsterones, L-tyrosine, iodine), cayenne & black pepper, and ginger root.

A significant amount of research has evaluated the safety and efficacy of EC and ECA type supplements. According to a meta-analysis in the Journal of American Medical Association, ephedrine/ephedra promote a more substantial weight loss 0.9 kg per month in comparison to placebo in clinical trials but are associated with increased risk of psychiatric, autonomic or gastrointestinal symptoms as well as heart palpitations. Several studies have confirmed that use of synthetic or herbal sources of ephedrine and caffeine (EC) promote about 2 lbs of extra weight loss per month while dieting (with or without exercise) and that EC supplementation is generally well tolerated in healthy individuals [263-274]. For example, Boozer et al [267] reported that 8-weeks of ephedrine (72 mg/d) and caffeine (240 mg/d) supplementation promoted a 9 lbs loss in body mass and a 2.1% loss in body fat with minor side effects. Hackman and associates [275] reported that a 9 month clinical trial utilizing a multi-nutrient supplement containing 40 mg/d of ephedra alkaloids and 100 mg/day caffeine resulted in a loss of weight and body fat, improved metabolic parameters including insulin sensitivity without any apparent side effects. Interestingly, Greenway and colleagues [274] reported that EC supplementation was a more cost-effective treatment for reducing weight, cardiac risk, and LDL cholesterol than several weight loss drugs (fenfluramine with mazindol or phentermine). Finally, Boozer and associates [268] reported that 6-months of herbal EC supplementation promoted weight loss with no clinically significant adverse effects in healthy overweight adults. Less is known about the safety and efficacy of synephrine, thyroid stimulators, cayenne/black pepper and ginger root.

Despite these findings, the Food and Drug Administration (FDA) banned the sale of ephedra containing supplements. The rationale has been based on reports to adverse event monitoring systems and in the media suggesting a link between intake of ephedra and a number of severe medical complications (e.g., high blood pressure, elevated heart rate, arrhythmias, sudden death, heat stroke, etc) [276,277]. Although results of available clinical studies do not show these types of adverse events, ephedra is no longer available as an ingredient in dietary supplements and thus cannot be recommended for use. Consequently, thermogenic supplements now contain other nutrients believed to increase energy expenditure (e.g., synephrine, green tea, etc) and are sold as "ephedrine-free" types of products. Anyone contemplating taking thermogenic supplements should carefully consider the potential side effects, discuss possible use with a knowledgeable physician, and be careful not to exceed recommended dosages.

#### Possibly Effective

##### High Fiber Diets

One of the oldest and most common methods of suppressing the appetite is to consume a diet that is high in fiber. Ingesting high fiber foods (fruits, vegetables) or fiber containing supplements (e.g., glucomannan) increase the feeling of fullness (satiety) which typically allows an individual to feel full while ingesting fewer calories. Theoretically, maintaining a high fiber diet may serve to help decrease the amount of food you eat. In addition, high fiber diets/supplements help lower cholesterol and blood pressure, enhance insulin sensitivity, and promote weight loss in obese subjects [278]. A recent study found that a Mediterranean diet that was high in fiber resulted in a more dramatic weight loss that a traditional low-fat diet and had beneficial effects on glycemic control [279]. Other research on high fiber diets indicates that they provide some benefit, particularly in diabetic populations. For example, Raben et al [280] reported that subjects maintaining a low fat/high fiber diet for 11 weeks lost about 3 lbs of weight and 3.5 lbs of fat. Other studies have reported mixed results on altering body composition using various forms of higher fiber diets [281-284]. Consequently, although maintaining a low fat/high fiber diet that is high in fruit and vegetable content has various health benefits, these diets seem to have potential to promote weight loss as well as weight maintenance thus we can recommend high fiber diets as a safe and healthy approach to possibly improve body composition.

##### Calcium

Several studies and recent reviews have reported that calcium supplementation alone or in combination with other ingredients does not affect weight loss or fat loss [285-290]. Research has indicated that calcium modulates 1,25-diydroxyvitamin D which serves to regulate intracellular calcium levels in fat cells [291,292]. Increasing dietary availability of calcium reduces 1,25-diydroxyvitamin D and promotes reductions in fat mass in animals [292-294]. Dietary calcium has been shown to suppress fat metabolism and weight gain during periods of high caloric intake [291,293,295]. Further, increasing calcium intake has been shown to increase fat metabolism and preserve thermogenesis during caloric restriction [291,293,295]. In support of this theory, Davies and colleagues [296] reported that dietary calcium was negatively correlated to weight and that calcium supplementation (1,000 mg/d) accounted for an 8 kg weight loss over a 4 yr period. Additionally, Zemel and associates [291] reported that supplemental calcium (800 mg/d) or high dietary intake of calcium (1,200 - 1,300 mg/d) during a 24-week weight loss program promoted significantly greater weight loss (26-70%) and dual energy x-ray absorptiometer (DEXA) determined fat mass loss (38-64%) compared to subjects on a low calcium diet (400-500 mg/d). These findings and others suggest a strong relationship between calcium intake and fat loss. However, more research needs to be conducted before definitive conclusions can be drawn.

##### Green Tea Extract

Green tea is now one of the most common herbal supplements that is being added to thermogenic products because it has been suggested to affect weight loss and is now the fourth most commonly used dietary supplement in the US [297]. Green tea contains high amounts of caffeine and catechin polyphenols. The primary catechin that is associated to the potential effects on weight loss through diet induced thermogenesis is the catechin epigallocatechin gallate, also known as EGCG [298,299]. Research suggests that catechin polyphenols possess antioxidant properties and the intake of tea catechins is associated with a reduced risk of cardiovascular disease [298-300]. In addition, green tea has also been theorized to increase energy expenditure by stimulating brown adipose tissue thermogenesis. In support of this theory, Dulloo et al [301,302] reported that green tea supplementation in combination with caffeine (e.g., 50 mg caffeine and 90 mg epigallocatechin gallate taken 3-times per day) significantly increased 24-hour energy expenditure and fat utilization in humans to a much greater extent than when an equivalent amount of caffeine was evaluated suggesting a synergistic effect. Recently, work by Di Pierro and colleagues [303] reported that the addition of a green tea extract to a hypocaloric diet resulted in a significant increase in weight loss (14 kg vs. 5 kg) versus a hypocaloric diet alone over a 90 day clinical trial. Maki and coworkers [304] also demonstrated that green tea catechin consumption enhanced the exercise-induced changes in abdominal fat. However, it must be noted that both human and animal studies have not supported these findings and have reported that supplementation of these extracts does not affect weight loss [305,306]. Theoretically, increases in energy expenditure may help individuals lose weight and/or manage body composition.

##### Conjugated Linoleic Acids (CLA)

CLA is a term used to describe a group of positional and geometric isomers of linoleic acid that contain conjugated double bonds. Adding CLA to the diet has been reported to possess significant health benefits in animals [184,307]. In terms of weight loss, CLA feedings to animals have been reported to markedly decrease body fat accumulation [185,308]. Consequently, CLA has been marketed as a health and weight loss supplement since the mid 1990s. Despite the evidence in animal models, the effect of CLA supplementation in humans is less clear. There are some data suggesting that CLA supplementation may modestly promote fat loss and/or increases in lean mass [190-192,309-314]. Recent work suggested that CLA supplementation coupled with creatine and whey protein resulted in a increase in strength and lean-tissue mass during resistance training [315]. However, other studies indicate that CLA supplementation (1.7 to 12 g/d for 4-weeks to 6-months) has limited to no effects on body composition alterations in untrained or trained populations [190,310,316-324]. The reason for the discrepancy in research findings has been suggested to be due to differences in purity and the specific isomer studied. For instance, early studies in humans showing no effect used CLA that contained all 24 isomers. Today, most labs studying CLA use 50-50 mixtures containing the trans-10, cis-12 and cis-9, trans-11 isomers, the former of which being recently implicated in positively altering body composition. This has been supported by recent work indicating that CLA (50:50 cis-9, trans-11:trans-10, cis-12) plus polyunsaturated fatty acid supplementation prevented abdominal fat increases and increase fat-free mass [325]. However, it must be noted that this response only occurred in young obese individuals. Thus, CLA supplementation may have potential in the areas of general health and it is clear that research on the effects on body composition is ongoing and still quite varied. Further research is needed to determine which CLA isomer is ideal for ingestion and possibly if there are differential responses among lean or obese and old or young populations.

#### Too Early to Tell

##### Gymnema Sylvestre

Gymnema Sylvestre is a supplement that is purported to regulate weight loss and blood sugar levels. It is purported to affect glucose and fat metabolism as well as inhibit sweet cravings. In support of these contentions, some recent data have been published by Shigematsu and colleagues [326,327] showing that short and long-term oral supplementation of gymnema sylvestre in rats fed normal and high-fat diets may have some positive effects on fat metabolism, blood lipid levels, and/or weight gain/fat deposition. More recent work in rats has shown that gymnema sylvestre supplementation promoted weight loss by reducing hyperlipidemia [328]. The only apparent clinical trial in humans showed that an herbal combination group containing 400 mg of gymnema sylvestra resulted in effective and safe weight loss while promoting improved blood lipid profiles. It should be noted that this group was not significantly different that the other active group, containing HCA, when observing these dependent variables [329]. Due to the lack of substantial positive research on the effects of gymnema sylvestre supplementation in humans, we cannot recommend gymnema sylvestre as a supplement to positively affect weight loss.

##### Phosphatidyl Choline (Lecithin)

Choline is considered an essential nutrient that is needed for cell membrane integrity and to facilitate the movement of fats in and out of cells. It is also a component of the neurotransmitter acetylcholine and is needed for normal brain functioning, particularly in infants. For this reason, phosphatidyl choline (PC) has been purported as a potentially effective supplement to promote fat loss as well as improve neuromuscular function. However, despite these alleged benefits of lecithin supplementation, there are no clinical trials in humans to support a potential role of lecithin supplementation affecting weight loss.

##### Betaine

Betaine is a compound that is involved in the metabolism of choline and homocysteine. Garcia Neto et al. [330] have shown that betaine feedings can effect liver metabolism, fat metabolism, and fat deposition in chickens. Betaine supplementation may also help lower homocysteine levels which is a marker of risk to heart disease [331]. For this reason, betaine supplements have been marketed as a supplement designed to promote heart health as well as a weight loss. A recent study by Hoffman and colleagues [332] found betaine supplementation to improve muscular endurance in active college age males. Despite this, there appears to be little evidence in human models that supports the role of betaine as a supplement for weight loss and thus it is not recommended for supplementation.

##### Coleus Forskohlii (Forskolin)

Forskolin, which is touted as a weight loss supplement is a plant native to India that has been used for centuries in traditional Ayurvedic medicine primarily to treat skin disorders and respiratory problems [333,334]. A considerable amount of research has evaluated the physiological and potential medical applications of forskolin over the last 25 years. Forskolin has been reported to reduce blood pressure, increase the hearts ability to contract, help inhibit platelet aggregation, improve lung function, and aid in the treatment of glaucoma [333-335]. With regard to weight loss, forskolin has been reported to increase cyclic AMP and thereby stimulate fat metabolism [336-338]. Theoretically, forskolin may therefore serve as an effective weight loss supplement. Recent evidence has shown that forskolin supplementation had no effect on improving body composition in mildly obese women [339]. In contrast, work done by Godard et al. in 2005 reported that 250 mg of a 10% forskolin extract taken twice daily resulted in improvements in body composition in overweight and obese men [340]. Another study suggested that supplementing the diet with coleus forskohlii in overweight women helped maintain weight and was not associated with any clinically significant adverse events [341]. Currently, research is still needed on forskolin supplementation before it can be recommended as an effective weight loss supplement.

##### Dehydroepiandrosterone (DHEA) and 7-Keto DHEA

Dehydroepiandrosterone (DHEA) and its sulfated conjugate DHEAS represent the most abundant adrenal steroids in circulation [342]. Although, DHEA is considered a weak androgen, it can be converted to the more potent androgens testosterone and dihydrotestosterone in tissues. In addition, DHEAS can be converted into androstenedione and testosterone. DHEA levels have been reported to decline with age in humans [343]. The decline in DHEA levels with aging has been associated with increased fat accumulation and risk to heart disease [344]. Since DHEA is a naturally occurring compound, it has been suggested that dietary supplementation of DHEA may help maintain DHEA availability, maintain and/or increase testosterone levels, reduce body fat accumulation, and/or reduce risk to heart disease as one ages [342,344]. Although animal studies have generally supported this theory, the effects of DHEA supplementation on body composition in human trials have been mixed. For example, Nestler and coworkers [345] reported that DHEA supplementation (1,600 mg/d for 28-d) in untrained healthy males promoted a 31% reduction in percentage of body fat. However, Vogiatzi and associates [346] reported that DHEA supplementation (40 mg/d for 8 wks) had no effect on body weight, percent body fat, or serum lipid levels in obese adolescents. More recent work has supported these findings suggesting that one year of DHEA supplementation had no effect on body composition when taken at 50 mg per day [347]. 7-keto DHEA, a DHEA precursor, has been marketed as a potentially more effective form of DHEA which is believed to possess lypolytic properties. Although data are limited, Kalman and colleagues and coworkers [348] reported that 7-keto DHEA supplementation (200 mg/d) during 8-weeks of training promoted a greater loss in body mass and fat mass while increasing T3 while observing no significant effects on thyroid stimulating hormone (TSH) or T4. More recent data has shown that 7-keto DHEA supplementation can increase RMR [349] and blunt the decrease in RMR associated with 8 weeks of restricted dieting [350]. However, it must be noted that the second study did not use isolated 7-keto DHEA but used a commercial weight loss product that contained DHEA as well as other known weight loss agents (i.e. caffeine, green tea extract, citrus aurantium, etc.). Thus, these results do not directly support the use of 7-keto DHEA. Although more research is needed on the effects of supplementing DHEA by itself as a weight loss agent, these findings provide minimal support that 7-keto DHEA may serve as an effective weight loss supplement.

##### Psychotropic Nutrients/Herbs

Psychotropic nutrients/herbs are a new class of supplements that often contain things like St. John's Wart, Kava, Ginkgo Biloba, Ginseng, and L-Tyrosine. They are believed to serve as naturally occurring antidepressants, relaxants, and mental stimulants thus the theoretical rationale regarding weight loss is that they may help people fight depression or maintain mental alertness while dieting. There are no clinical weight loss trials that utilize any of the above nutrients/herbs as the active ingredient in the supplementation trial. Although a number of studies support potential role as naturally occurring psychotropics or stimulants, the potential value in promoting weight loss is unclear and therefore are not recommended for supplementation.

#### Apparently Ineffective

##### Calcium Pyruvate

Calcium Pyruvate is supplement that hit the scene about 10-15 ago with great promise. The theoretical rationale was based on studies from the early 1990s that reported that calcium pyruvate supplementation (16 - 25 g/d with or without dihydroxyacetone phosphate [DHAP]) promoted fat loss in overweight/obese patients following a medically supervised weight loss program [351-353]. Although the mechanism for these findings was unclear, the researchers speculated that it might be related to appetite suppression and/or altered carbohydrate and fat metabolism. Since calcium pyruvate is very expensive, several studies have attempted to determine whether ingesting smaller amounts of calcium pyruvate (6-10 g/d) affect body composition in untrained and trained populations. Results of these studies are mixed. Earlier studies have shown both a positive effect on calcium pyruvate supplementation in improving body composition [354], however, Stone and colleagues [355] reported that pyruvate supplementation did not affect hydrostatically determined body composition during 5-weeks of in-season college football training. More recently, calcium pyruvate supplementation was also shown to not have a significant effect on body composition or exercise performance. Additionally, it has been reported that supplementation may negatively affect some blood lipid levels [356]. These findings indicate that although there is some supportive data indicating that calcium pyruvate supplementation may enhance fat loss when taken at high doses (6-16 g/d), there is no evidence that ingesting the doses typically found in pyruvate supplements (0.5 - 2 g/d) has any affect on body composition. In addition, the overall quantity of research examining calcium pyruvate is minimal at best thus it is not warranted to include calcium pyruvate as a weight loss supplement.

##### Chitosan

Chitosan has been marketed as a weight loss supplement for several years as is known as a "fat trapper". It is purported to inhibit fat absorption and lower cholesterol. This notion is supported animal studies indicated by decreased fat absorption, increased fat content, and/or lower cholesterol following chitosan feedings [357-360]. However, the effects in humans appear to be less impressive. For example, although there is some data suggesting that chitosan supplementation may lower blood lipids in humans,[361] other studies report no effects on fat content [362,363]or body composition alterations [364-366] when administered to people following their normal diet. More recent work has shown that the effect of chitosan on fat absorption is negligible and is the equivalent of approximately 9.9 kcal/day following supplementation [362]. Other work has concluded that the insignificant amounts of fat that are trapped from supplementation would take about 7 months for a male to lose a pound of weight, and that the effect was completely ineffective in women [364]. Thus, based on the current evidence, chitosan supplementation is apparently ineffective and has no significant effects on "fat trapping" and/or on improving body composition.

##### Chromium

Chromium supplementation is derived from its role in maintaining proper carbohydrate and fat metabolism by potentially effecting insulin signalling [367]. Initial studies reported that chromium supplementation during resistance training improved fat loss and gains in lean body mass [173-175]. To date, the studies using more accurate methods of assessing body composition have primarily indicate no effects on body composition in healthy non-diabetic individuals [176-183,368]. Recent work has reported that 200 mcg of chromium picolinate supplementation on individuals on a restrictive diet did not promote weight loss or body composition changes following 12 weeks of supplementation [368]. This work supports Lukaski et al [182] previous findings that 8-weeks of chromium supplementation during resistance training did not affect strength or DEXA determined body composition changes. Thus, based on the current review of the literature we cannot recommend chromium supplementation as a means of improving body composition.

##### Garcinia Cambogia (HCA)

HCA is a nutrient that has been hypothesized to increase fat oxidation by inhibiting citrate lypase and lipogenesis [369]. Theoretically, this may lead to greater fat burning and weight loss over time. Although there is some evidence that HCA may increase fat metabolism in animal studies, there is little to no evidence showing that HCA supplementation affects body composition in humans. For example, Ishihara et al [370] reported that HCA supplementation spared carbohydrate utilization and promoted lipid oxidation during exercise in mice. However, Kriketos and associates [371] reported that HCA supplementation (3 g/d for 3-days) did not affect resting or post-exercise energy expenditure or markers of lipolysis in healthy men. Likewise, Heymsfield and coworkers [372] reported that HCA supplementation (1.5 g/d for 12-weeks) while maintaining a low fat/high fiber diet did not promote greater weight or fat loss than subjects on placebo. Finally, Mattes and colleagues [373] reported that HCA supplementation (2.4 g/d for 12-weeks) did not affect appetite, energy intake, or weight loss. These findings suggest that HCA supplementation does not appear to promote fat loss in humans.

##### L-Carnitine

Carnitine serves as an important transporter of fatty acids from the cytosol into the mitochondria of the cell [374]. Increased cellular levels of carnitine would theoretically enhance transport of fats into the mitochondria and thus provide more substrates for fat metabolism. L-carnitine has been one of the most common nutrients found in various weight loss supplements. Over the years, a number of studies have been conducted on the effects of L-carnitine supplementation on fat metabolism, exercise capacity and body composition. The overwhelming conclusions of L-carnitine research indicates that L-carnitine supplementation does not affect muscle carnitine content [375], fat metabolism, aerobic- or anaerobic-exercise performance [375], and/or weight loss in overweight or trained subjects [376,377]. Despite the fact that L-carnitine has been shown apparently ineffective as a supplement, the research on L-carnitine has shifted to another category revolving around hypoxic stress and oxidative stress. Preliminary research has reported that L-carnitine supplementation has a minimal effect on reducing the biomarkers of exercise-induced oxidative stress [378]. While these findings are not promising, there is some recent data indicating that L-carnitine tartrate supplementation during intensified periods of training may help athletes tolerate training to a greater degree [379]. Consequently, there may be other advantages to L-carnitine supplementation than promoting fat metabolism.

##### Phosphates

The role of sodium and calcium phosphate on energy metabolism and exercise performance has been studied for decades [31]. Phosphate supplementation has also been suggested to affect energy expenditure, however, the research in this area is quite dated and no research on the effects on energy expenditure have been conducted. Some of this dated work includes the work by Kaciuba-Uscilko and colleagues [380] who reported that phosphate supplementation during a 4-week weight loss program increased resting metabolic rate (RMR) and respiratory exchange ratio (suggesting greater carbohydrate utilization and caloric expenditure) during submaximal cycling exercise. In addition, Nazar and coworkers [381] reported that phosphate supplementation during an 8-week weight loss program increased RMR by 12-19% and prevented a normal decline in thyroid hormones. Although the rate of weight loss was similar in this trial, results suggest that phosphate supplementation may influence metabolic rate possibly by affecting thyroid hormones. Despite these to dated trials, no further research has been conducted and thus the role of phosphates in regards to weight loss is inconclusive at best.

##### Herbal Diuretics

This is a new type of supplement recently marketed as a natural way to promote weight loss. There is limited evidence that taraxacum officinale, verbena officinalis, lithospermum officinale, equisetum arvense, arctostaphylos uva-ursi, arctium lappa and silene saxifraga infusion may affect diuresis in animals [382,383]. Two studies presented at the 2001 American College of Sports Medicine meeting [384,385] indicated that although herbal diuretics promoted a small amount of dehydration (about 0.3% in one day), they were not nearly as effective as a common diuretic drug (about 3.1% dehydration in one day). Consequently, although more research is needed, the potential value of herbal diuretics as a weight loss supplement appears limited.

### Performance Enhancement Supplements

A number of nutritional supplements have been proposed to enhance exercise performance. Some of these nutrients have been described above. Table 3 categorizes the proposed ergogenic nutrients into apparently safe and effective, possibly effective, too early to tell, and apparently ineffective. Weight gain supplements purported to increase muscle mass may also have ergogenic properties if they also promote increases in strength. Similarly, some sports may benefit from reductions in fat mass. Therefore, weight loss supplements that help athletes manage body weight and/or fat mass may also possess some ergogenic benefit. The following describes which supplements may or may not affect performance that were not previously described.

#### Apparently Effective

##### Water and Sports Drinks

Preventing dehydration during exercise is one of the keys of maintaining exercise performance (particularly in hot/humid environments). People engaged in intense exercise or work in the heat need to frequently ingest water or sports drinks (e.g., 1-2 cups every 10 - 15 minutes). The goal should be not to lose more than 2% of body weight during exercise (e.g., 180 lbs × 0.02 = 3.6 lbs). Sports drinks typically contain salt and carbohydrate at scientifically engendered quantities. Studies show that ingestion of sports drinks during exercise in hot/humid environments can help prevent dehydration and improve endurance exercise capacity [[56], von Duvillard 2005), [386,387]]. In fact, research has shown that carbohydrate intake during team sport type activities can increase exercise performance and CNS function [15,16,388]. Consequently, frequent ingestion of water and/or sports drinks during exercise is one of the easiest and most effective ergogenic aids.

##### Carbohydrate

One of the best ergogenic aids available for athletes and active individuals alike, is carbohydrate. Athletes and active individuals should consume a diet high in carbohydrate (e.g., 55 - 65% of calories or 5-8 grams/kg/day) in order to maintain muscle and liver carbohydrate stores [1,3]. Research has clearly identified carbohydrate is an ergogenic aid that can prolong exercise [3]. Additionally, ingesting a small amount of carbohydrate and protein 30-60 minutes prior to exercise and use of sports drinks during exercise can increase carbohydrate availability and improve exercise performance. Finally, ingesting carbohydrate and protein immediately following exercise can enhance carbohydrate storage and protein synthesis [1,3].

##### Creatine

Earlier we indicated that creatine supplementation is one of the best supplements available to increase muscle mass and strength during training. However, creatine has also been reported to improve exercise capacity in a variety of events [[71], Kendall 2005, [389-391]]. This is particularly true when performing high intensity, intermittent exercise such as multiple sets of weight lifting, repeated sprints, and/or exercise involving sprinting and jogging (e.g., soccer) [71]. Creatine has also been shown to be effective at improving high intensity interval training. A 2009 study found that in addition to high intensity interval training creatine improved critical power [390]. Although studies evaluating the ergogenic value of creatine on endurance exercise perfor mance are mixed, endurance athletes may also theoretically benefit in several ways. For example, increasing creatine stores prior to carbohydrate loading (i.e., increasing dietary carbohydrate intake before competition in an attempt to maximize carbohydrate stores) has been shown to improve the ability to store carbohydrate [392-394]. A 2003 study found that ingesting 20 grams of creatine for 5 days improved endurance and anaerobic performance in elite rowers [395]. Further, co ingesting creatine with carbohydrate has been shown to optimize creatine and carbohydrate loading [396]. Most endurance athletes also perform interval training (sprint or speed work) in an attempt to improve anaerobic threshold. Since creatine has been reported to enhance interval sprint performance, creatine supplementation during training may improve training adaptations in endurance athletes [397,398]. Finally, many endurance athletes lose weight during their competitive season. Creatine supplementation during training may help people maintain weight.

##### Sodium Phosphate

We previously mentioned that sodium phosphate supplementation may increase resting energy expenditure and therefore could serve as a potential weight loss nutrient. However, most research on sodium phosphate has actually evaluated the potential ergogenic value. A number of studies indicated that sodium phosphate supplementation (e.g., 1 gram taken 4 times daily for 3-6 days) can increase maximal oxygen uptake (i.e., maximal aerobic capacity) and anaerobic threshold by 5-10% [399-403]. These finding suggest that sodium phosphate may be highly effective in improving endurance exercise capacity. In addition to endurance enhancement, sodium phosphate loading improved mean power output and oxygen uptake in trained cyclist in a 2008 study [404]. Other forms of phosphate (i.e., calcium phosphate, potassium phosphate) do not appear to possess ergogenic value.

##### Sodium Bicarbonate (Baking Soda)

During high intensity exercise, acid (H+) and carbon dioxide (CO_2_) accumulate in the muscle and blood. One of the ways you get rid of the acidity and CO_2 _is to buffer the acid and CO_2 _with bicarbonate ions. The acid and CO_2 _are then removed in the lungs. Bicarbonate loading (e.g., 0.3 grams per kg taken 60-90 minutes prior to exercise or 5 grams taken 2 times per day for 5-days) has been shown to be an effective way to buffer acidity during high intensity exercise lasting 1-3 minutes in duration [405-408]. This can improve exercise capacity in events like the 400 - 800 m run or 100 - 200 m swim [409]. In elite male swimmers sodium bicarbonate supplementation significantly improved 200 m freestyle performance [410]. A 2009 study found similar improvements in performance in youth swimmers at distances of 50 to 200 m. Although bicarbonate loading can improve exercise, some people have difficulty with their stomach tolerating bicarbonate as it may cause gastrointestinal distress.

##### Caffeine

Caffeine is a naturally derived stimulant found in many nutritional supplements typically as gaurana, bissey nut, or kola. Caffeine can also be found in coffee, tea, soft drinks, energy drinks, and chocolate. It has previously been made clear that caffeine can have a positive effect on energy expenditure, weight loss, and body fat. Caffeine has also been shown to be an effective ergogenic aid. Research investigating the effects of caffeine on a time trial in trained cyclist found that caffeine improved speed, peak power, and mean power [411]. Similar results were observed in a recent study that found cyclists who ingested a caffeine drink prior to a time trial demonstrated improvements in performance [412,413]. Studies indicate that ingestion of caffeine (e.g., 3-9 mg/kg taken 30 - 90 minutes before exercise) can spare carbohydrate use during exercise and thereby improve endurance exercise capacity [406,414]. In addition to the apparent positive effects on endurance performance, caffeine has also been shown to improve repeated sprint performance benefiting the anaerobic athlete [415,416]. People who drink caffeinated drinks regularly, however, appear to experience less ergogenic benefits from caffeine [417]. Additionally, some concern has been expressed that ingestion of caffeine prior to exercise may contribute to dehydration although recent studies have not supported this concern [414,418,419]. Caffeine doses above 9 mg/kg can result in urinary caffeine levels that surpass the doping threshold for many sport organizations. Suggestions that there is no ergogenic value to caffeine supplementation is not supported by the preponderance of available scientific studies.

##### β-alanine

In recent years research has begun investigating the effects of β-alanine supplementation on performance. β-alanine has ergogenic potential based on its relationship with carnosine. Carnosine is a dipeptide comprised of the amino acids, histidine and β-alanine naturally occurring in large amounts in skeletal muscles. Carnosine is believed to be one of the primary muscle-buffering substances available in skeletal muscle. Studies have demonstrated that taking β-alanine orally over a 28-day period was effective in increasing carnosine levels [420,421]. This proposed benefit would increase work capacity and decrease time to fatigue. Researchers have found that β-alanine supplementation decreases rate of fatigue [422]. This could translate into definite strength gains and improved performance. A recent study [423] supplemented men with β-alanine for 10 weeks and showed that muscle carnosine levels were significantly increased after 4 and 10 weeks of β-alanine supplementation.

Stout et al. [422] conducted a study that examined the effects of β-alanine supplementation on physical working capacity at fatigue threshold. The results showed decreased fatigue in the subjects tested. Other studies have shown that β-alanine supplementation can increase the number of repetitions one can do [424], increased lean body mass [425], increase knee extension torque [426] and training volume [427]. In fact, one study also showed that adding β-alanine supplementation with creatine improves performance over creatine alone [428]. While it appears that β-alanine supplementation can decrease fatigue rate, raise carnosine levels, and improve performance all of the research is not as favorable. There are other studies that show no performance benefits [425,429]

#### Possibly Effective

##### Post-Exercise Carbohydrate and Protein

Ingesting carbohydrate and protein following exercise enhances carbohydrate storage and protein synthesis. Theoretically, ingesting carbohydrate and protein following exercise may lead to greater training adaptations. In support of this theory, Esmarck and coworkers [107] found that ingesting carbohydrate and protein immediately following exercise doubled training adaptations in comparison to waiting until 2-hours to ingest carbohydrate and protein. Additionally, Tarnopolsky and associates [430] reported that post-exercise ingestion of carbohydrate with protein promoted as much strength gains as ingesting creatine with carbohydrate during training. A recent study by Kreider and colleagues [431] found that protein and carbohydrate supplementation post workout was capable of positively supporting the post exercise anabolic response. In the last few years many studies have agreed with these findings in that post workout supplementation is vital to recovery and training adaptations [13,104,431-433]. These findings underscore the importance of post-exercise carbohydrate and protein ingestion to support muscle anabolism and strength. However, it is still unclear if there are direct implications of protein/carbohydrate supplementation on other markers of performance such as time to exhaustion, maximal oxygen uptake, and/or skill development.

##### Essential Amino Acids (EAA)

Ingestion of 3-6 grams of EAA following resistance exercise has been shown to increase protein synthesis [92,93,98-102,105,434]. Theoretically, ingestion of EAA after exercise should enhance gains in strength and muscle mass during training. While there is sound theoretical rationale, it is currently unclear whether following this strategy would lead to greater training adaptations and/or whether EAA supplementation would be better than simply ingesting carbohydrate and a quality protein following exercise.

##### Branched Chain Amino Acids (BCAA)

Ingestion of BCAA (e.g., 6-10 grams per hour) with sports drinks during prolonged exercise would theoretically improve psychological perception of fatigue (i.e., central fatigue). Although there is strong rationale, the effects of BCAA supplementation on exercise performance is mixed with some studies suggesting an improvement and others showing no effect [33]. More research is needed before conclusions can be drawn.

##### β-HMB

HMB supplementation has been reported to improve training adaptations in untrained individuals initiating training as well as help reduce muscle breakdown in runners. Theoretically, this should enhance training adaptations in athletes. However, most studies show little benefit of HMB supplementation in athletes. A 2004 study by Hoffman [435] found HMB supplementation to be ineffective in collegiate football players after short term supplementation. It has been hypothesized that HMB will delay or prevent muscle damage; however this has limited evidence as suggested in previous sections. There are a few studies that have been positive [115]. A 2009 study found that HMB *supplementation *did positively affect strength in trained men [436]. While HMB supplementation may still have some scientific rationale there is little evidence that is can directly affect performance in moderately trained subjects.

##### Glycerol

Ingesting glycerol with water has been reported to increase fluid retention [437]. Theoretically, this should help athletes prevent dehydration during prolonged exercise and improve performance particularly if they are susceptible to dehydration. Although studies indicate that glycerol can significantly enhance body fluid, results are mixed on whether it can improve exercise capacity [69,438-443]. Little research has been done on glycerol in the last five years however, a 2006 study agreed with previous findings in that glycerol has little impact on performance [444].

#### Too Early to Tell

A number of supplements purported to enhance performance and/or training adaptation fall under this category. This includes the weight gain and weight loss supplements listed in Table 3 as well as the following supplements not previously described in this category.

##### Medium Chain Triglycerides (MCT)

MCT's are shorter chain fatty acids that can easily enter the mitochondria of the cell and be converted to energy through fat metabolism [445]. Studies are mixed as to whether MCT's can serve as an effective source of fat during exercise metabolism and/or improve exercise performance [445-449]. A 2001 study found that 60 g/day of MCT oil for two weeks was not sufficient at improving performance [450]. In fact Goedecke found that not only did MCT supplementation not improve performance, but, actually negatively affected sprint performance in trained cyclists [451]. These findings have been confirmed by others that MCT oils are not sufficient to induce positive training adaptations and may cause gastric distress [452,453]. It must be noted that while most studies have not been favourable, one 2009 study found that MCT oil may positively affect RPE and lactate clearance [454]. It does not appear likely that MCT can positively affect training adaptations, but further research is needed.

#### Apparently Ineffective

##### Glutamine

As described above, glutamine has been shown to influence protein synthesis and help maintain the immune system. Theoretically, glutamine supplementation during training should enhance gains in strength and muscle mass as well as help athletes tolerate training to a better degree. Although there is some evidence that glutamine supplementation with protein can improve training adaptations, more research is needed to determine the ergogenic value in athletes. There is currently no research to suggest that glutamine has a direct effect on performance.

##### Ribose

Ribose is a 3-carbon carbohydrate that is involved in the synthesis of adenosine triphosphate (ATP) in the muscle (the useable form of energy). Clinical studies have shown that ribose supplementation can increase exercise capacity in heart patients [455-459]. For this reason, ribose has been suggested to be an ergogenic aid for athletes. Although more research is needed, most studies show no ergogenic value of ribose supplementation on exercise capacity in health untrained or trained populations [460-462]. A 2006 study [463] investigated the effects of ribose vs. dextrose on rowing performance. After eight weeks of supplementation dextrose had a better response than ribose across the subjects [463]. Kreider and associates [462] and Kersick and colleagues [464] investigated ribose supplementation on measures of anaerobic capacity in trained athletes. This research group found that ribose supplementation did not have a positive impact on performance [462,464]. It appears at this point that ribose supplementation does not improve aerobic or anaerobic performance.

##### Inosine

Inosine is a building block for DNA and RNA that is found in muscle. Inosine has a number of potentially important roles that may enhance training and/or exercise performance [465]. Although there is some theoretical rationale, available studies indicate that inosine supplementation has no apparent affect on exercise performance capacity [466-468].

### Supplements to Promote General Health

In addition to the supplements previously described, several nutrients have been suggested to help athletes stay healthy during intense training. For example, the American Medical Association recently recommended that all Americans ingest a daily low-dose multivitamin in order to ensure that people get a sufficient amount of vitamins and minerals in their diet. Although one-a-day vitamin supplementation has not been found to improve exercise capacity in athletes, it may make sense to take a daily vitamin supplement for health reasons. Glucosomine and chondroitin have been reported to slow cartilage degeneration and reduce the degree of joint pain in active individuals which may help athletes postpone and/or prevent joint problems [469,470]. Supplemental Vitamin C, glutamine, echinacea, and zinc have been reported to enhance immune function [471-474]. Consequently, some sports nutritionists recommend that athletes who feel a cold coming on take these nutrients in order to enhance immune function [55,471-473]. Similarly, although additional research is necessary, Vitamin E, Vitamin C, selenium, alpha-lipoic acid and other antioxidants may help restore overwhelmed anti-oxidant defences exhibited by athletes and reduce the risk of numerous chronic diseases in some instances [475]. Creatine, calcium β-HMB, BCAA, and L-carnitine tartrate have been shown to help athletes tolerate heavy training periods [31,118,125,126,128,379,476-478]. Finally, the omega-3 fatty acids docosahexaenoic acid (DHA) and eicosapantaenoic acid (EPA), in supplemental form, are now endorsed by the American Heart Association for heart health in certain individuals [479]. This supportive supplement position stems from: 1.) an inability to consume cardio-protective amounts by diet alone; and, 2.) the mercury contamination sometimes present in whole-food sources of DHA and EPA found in fatty fish. Consequently, prudent use of these types of nutrients at various times during training may help athletes stay healthy and/or tolerate training to a greater degree [50].

## Conclusion

Maintaining an energy balance and nutrient dense diet, prudent training, proper timing of nutrient intake, and obtaining adequate rest are the cornerstones to enhancing performance and/or training adaptations. Use of a limited number of nutritional supplements that research has supported can help improve energy availability (e.g., sports drinks, carbohydrate, creatine, caffeine, β-alanine, etc) and/or promote recovery (carbohydrate, protein, essential amino acids, etc) can provide additional benefit in certain instances. The sports nutrition specialist should stay up to date regarding the role of nutrition on exercise so they can provide honest and accurate information to their students, clients, and/or athletes about the role of nutrition and dietary supplements on performance and training. Furthermore, the sports nutrition specialist should actively participate in exercise nutrition research; write unbiased scholarly reviews for journals and lay publications; help disseminate the latest research findings to the public so they can make informed decisions about appropriate methods of exercise, dieting, and/or whether various nutritional supplements can affect health, performance, and/or training; and, disclose any commercial or financial conflicts of interest during such promulgations to the public. Finally, companies selling nutritional supplements should develop scientifically based products, conduct research on their products, and honestly market the results of studies so consumers can make informed decisions.

## Competing interests

Authors of this paper have not received any financial remuneration for preparing or reviewing this paper. However, in an interest of full-disclosure as recommended in this paper, authors report the following competing interests. RBK has received university-funded grants to conduct research on several nutrients discussed in this paper and currently receives research funding from Curves International, General Mills Bell Institute for Human Nutrition; and, the National Institutes of Health. In addition, he has served as a paid consultant for industry; is currently serving as a product development consultant for Supreme Protein, has received honoraria for speaking at conferences and writing lay articles about topics discussed in this paper; receives royalties from the sale of several exercise and nutrition-related books; and, has served as an expert witness on behalf of the plaintiff and defense in cases involving dietary supplements. CW has received academic and industry funding related to dietary supplements and honoraria from speaking engagements on the topic. LT has received academic and industry funding related to dietary supplements and honoraria from speaking engagements on the topic. BC has received university and private sector funded grants to conduct research on several nutrients discussed in this paper and has received compensation for speaking at conferences and writing lay articles/books about topics discussed in this paper. ALA has received consulting fees from AquaGenus, Bergstrom Nutrition, Bioiberica, Curves International, Indena, Indfrag, Miami Research Associates, Omniactives, Sabinsa, and Yor Health; received dietary ingredient materials from Alzchem, Glanbia, and Lonza; sits on the board of New Era; has executive positions in Fein Innovations, Fierce Foods, and GENr8; has equity in AquaGenus, Fein Innovations, Fierce Foods, and GENr8; has stock options in New Era Nutrition and Scientific Food Solutions; has received royalties from Isatori; is a lead inventor on a patent pending related to vitamin K and MSM; has received travel and lodging reimbursement from Bergstrom, Danisco, Indfrag, and New Era Nutrition; has received in-kind compensation from Advanced Research Press; and is on the editorial advisory board of *Nutrition Business Journal*, and is a columnist for *Nutraceuticals World *and *Muscular Development*. RC is the attorney for numerous companies in the dietary supplement industry and has received payment for consultancy and the writing of lay articles discussing nutritional supplements. MC has served as a consultant for industry and received honoraria for speaking about topics discussed in this paper. CPE received honoraria from scientific and lay audience speaking engagements; has served as an expert witness for several patent litigations involving dietary supplements on the behalf of the plaintiff and defense; and, currently has a grant from the Gatorade Sports Science Institute involving the examination of a dietary supplement and its effect on athletic performance. MG has received academic and industry funding to conduct sport/exercise nutritional supplement research; has served as a paid consultant for the sports nutrition industry; and, has received honoraria for speaking engagements and publishing articles in lay sport nutrition venues. DSK has received grants and contracts to conduct research on several nutrients discussed in this paper; has served as a paid consultant for industry; has received honoraria for speaking at conferences and writing lay articles about topics discussed in this paper; receives royalties from the sale of several exercise and nutrition-related books; and, has served as an expert witness on behalf of the plaintiff and defense in cases involving dietary supplements. CMK has received academic and industry funding related to dietary supplements and honoraria from speaking engagements on the topic. In addition, he has received payment for writing of lay articles discussing nutritional supplements. SMK has served as a paid consultant for industry; has received honoraria for speaking at conferences and writing lay articles about topics discussed in this paper; receives royalties from the sale of several exercise and nutrition related books; and, receives commission and has stock in companies that sell products produced from several ingredients discussed in this paper. HL reports having received honoraria for lectures from scientific, educational and community groups; serving as a consultant and scientific advisory board member for Nordic Naturals, Inc.; payment for scientific and technical writing for Optimal Aging and Aesthetic Medicine, LLC.; payment for commercial writing for Essentials of Healthy Living; consultancy fees as owner of Physicians Pioneering Performance, LLC.; owner and medical director of Performance Spine and Sports Medicine, LLC.; and, owner and medical director of Northeast Spine and Sports Medicine, PC. LML has received academic and industry funding related to dietary supplements and honoraria from speaking engagements on the topic and has received payment for consultancy and the writing of lay articles discussing nutritional supplements. RM has received industry funding and stock options related to dietary supplement research. RM has also received honoraria for speaking and payment for consultancy and the writing of lay articles on nutritional supplements. AS reports no competing interests. MS has received honoraria from academic organizations for speaking at conferences and writing lay articles on various sports nutrition topics. TNZ has received university and contract research organization-funded grants to conduct research on several ingredients discussed in this paper; has served as a paid consultant for the sports nutrition industry; has received honoraria for speaking at conferences and writing lay articles about topics discussed in this paper; has received royalties from the sale of dietary supplements; has stock in a company that sells several ingredients discussed in this paper; and, has served as an expert witness in cases involving dietary supplements. RW has received industry funds for consultancy and employment related to dietary supplement development and marketing. DSW has received university and contract research organization-funded grants to conduct research on several ingredients discussed in this paper. He has previously served as a paid consultant for the nutraceutical and sports nutrition industry with the companies, Amino Vital and Transformation Enzyme, and is presently a paid consultant for VPX. He has received honoraria for speaking at conferences and writing lay articles about topics discussed in this paper. JA is the CEO of the ISSN and has received academic and industry (i.e. VPX/Redline) funding related to dietary supplement consultation, speaking engagements and writing on the topic.

## Authors' contributions

RBK contributed most of the content and served as senior editor of the paper. CDW, LT, and BC updated references, updated several sections of the paper, and assisted in editing content. ALA, RC, MC, CPE, MG, DSK, CMK, SMK, BL, HL, LML, RM, AS, MS, RW, DSW, TNZ, and JA reviewed and edited the manuscript. All authors read and approved the final manuscript.
